# 3D Hierarchical Sunflower‐Shaped MoS_2_/SnO_2_ Photocathodes for Photo‐Rechargeable Zinc Ion Batteries

**DOI:** 10.1002/advs.202309555

**Published:** 2024-03-19

**Authors:** Xinyang Wen, Yaotang Zhong, Shuai Chen, Zhengchi Yang, Pengyu Dong, Yuqi Wang, Linghai Zhang, Zhen Wang, Yue Jiang, Guofu Zhou, Junming Liu, Jinwei Gao

**Affiliations:** ^1^ Institute for Advanced Materials and Guangdong Provincial Key Laboratory of Quantum Engineering and Quantum Materials South China Academy of Advanced Optoelectronics South China Normal University Guangzhou 510006 China; ^2^ School of Chemistry South China Normal University Guangzhou 510006 China; ^3^ School of Flexible Electronics (Future Technologies) Nanjing Tech University Nanjing 211816 China; ^4^ Guangdong Provincial Key Laboratory of Optical Information Materials and Technology & Institute of Electronic Paper Displays South China Academy of Advanced Optoelectronics South China Normal University Guangzhou 510006 China; ^5^ Laboratory of Solid State Microstructures Nanjing University Nanjing 210093 China; ^6^ Centre for Advanced Optoelectronics School of Physics and Electronic Information Gannan Normal University Ganzhou Jiangxi 341000 China

**Keywords:** 3D Hierarchical, MoS_2_/SnO_2_ Photocathodes, Photo‐Rechargeable Zinc Ion Batteries

## Abstract

Photo‐rechargeable zinc‐ion batteries (PRZIBs) have attracted much attention in the field of energy storage due to their high safety and dexterity compared with currently integrated lithium‐ion batteries and solar cells. However, challenges remain toward their practical applications, originating from the unsatisfactory structural design of photocathodes, which results in low photoelectric conversion efficiency (PCE). Herein, a flexible MoS_2_/SnO_2_‐based photocathode is developed via constructing a sunflower‐shaped light‐trapping nanostructure with 3D hierarchical and self‐supporting properties, enabled by the hierarchical embellishment of MoS_2_ nanosheets and SnO_2_ quantum dots on carbon cloth (MoS_2_/SnO_2_ QDs@CC). This structural design provides a favorable pathway for the effective separation of photogenerated electron‐hole pairs and the efficient storage of Zn^2+^ on photocathodes. Consequently, the PRZIB assembled with MoS_2_/SnO_2_ QDs@CC delivers a desirable capacity of 366 mAh g^−1^ under a light intensity of 100 mW cm^−2^, and achieves an ultra‐high PCE of 2.7% at a current density of 0.125 mA cm^−2^. In practice, an integrated battery system consisting of four series‐connected quasi‐solid‐state PRZIBs is successfully applied as a wearable wristband of smartwatches, which opens a new door for the application of PRZIBs in next‐generation flexible energy storage devices.

## Graphical abstract

A flexible photocathode for photo‐rechargeable zinc ion batteries is developed by the hierarchical embellishment of sunflower‐shaped MoS_2_ and SnO_2_ quantum dots on carbon cloth (MoS_2_/SnO_2_ QDs@CC), in which the highly photosensitive MoS_2_ facilitates light absorption and the SnO_2_ quantum dots accelerate electron transport, resulting in a fast photoelectric response.

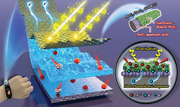

## 1 Introduction

Photovoltaic technology (PV) shows a fantastic application prospect in energy systems due to the absolute advantages of environmental friendliness and renewable resources.^[^ ^1^, ^2^, ^3^ ^]^ Nowadays, solar cells with silicon modules have been widely applied in large‐scale power grids. Although the solar cell is capable of efficiently harvesting and converting solar energy into electric power, it requires external energy devices for the storage of electricity, leading to complex system configurations and high operating costs.^[^ ^4^, ^5^, ^6^ ^]^ Therefore, the design and development of solar‐driven energy systems integrating electricity generation and storage are highly desirable yet greatly challenging.^[^ ^7^, ^8^, ^9^, ^10^, ^11^, ^12^, ^13^ ^]^

In recent years, considerable efforts have been made to integrate the dual functions of photoelectric conversion and electrochemical energy storage into the structures of photo‐charging integrated devices, which is a promising strategy for achieving cost‐effective and highly efficient photo‐rechargeable energy systems.^[^ ^14^, ^15^ ^]^ Generally, during the process of photo‐charging, the photo‐electrons and holes excited by illumination promote the oxide reaction on the photocathodes and the redox reaction on anodes, respectively, thereby successfully achieving the conversion of solar energy into electrochemical energy in battery systems.^[^ ^16^, ^17^, ^18^, ^19^ ^]^ To date, some successful experiences have been accumulated in the fields of photo‐rechargeable batteries especially lithium‐ion batteries (LIBs), lithium‐sulfur batteries (LSBs), and sodium‐ion batteries (SIBs).^[^ ^18^, ^20^, ^21^, ^22^, ^23^, ^24^, ^25^, ^26^, ^27^, ^28^, ^29^, ^30^, ^31^, ^32^, ^33^, ^34^, ^35^ ^]^ However, these battery systems still suffer from high manufacturing costs, low operational safety, and harsh production conditions, limiting their large‐scale application.^[^ ^36^ ^]^

Attractively, metal zinc (Zn) possesses the desirable advantages of natural abundance, low cost, intrinsic safety, high theoretical capacity (820 mAh g^−1^), and low redox potential (−0.762 V vs standard hydrogen electrode), showing a brighter future for battery systems.^[^ ^29^, ^37^, ^38^, ^39^ ^]^ Photo‐rechargeable Zn ion batteries (PRZIBs) combining photocathodes with Zn anodes are expected for the applied in large‐scale solar‐driven energy systems and are also considered to be an ideal energy storage for miniature wearable electronics in the future.^[^ ^7^ ^]^ So far, many 2D‐planar photocathodes with high‐photoactive materials have been employed in PRZIBs, such as vanadium oxides (VO_2_, V_2_O_5_)^[^ ^30^, ^31^, ^32^ ^]^ and molybdenum disulfide (MoS_2_).^[^ ^33^, ^40^ ^]^ For example, Boruah et al. in 2020^[^ ^31^ ^]^ first reported a PRZIB photocathode comprising a mixture of vanadium oxide (V_2_O_5_) nanofibers, P3HT and reduced graphene oxide (rGO) dropped on carbon cloth (CC) substrate, which demonstrated a photoelectric conversion efficiency (PCE) of 1.2% and delivered a capacity of 370 mAh g^−1^ at 50 mA g^−1^ under light source (λ ≈ 455 nm, intensity ≈ 12 mW cm^−2^). However, the physical random mixing of photosensitive materials with conductive agents and polymeric binders would reduce their active sites on photocathodes, resulting in the low PCE of PRZIBs. Thereafter, Boruah's work in 2021^[^ ^33^ ^]^ reported a structurally improved photocathode by synthesizing a zinc oxide (ZnO) film directly on the CC substrate and followed by in situ deposition of molybdenum disulfide (MoS_2_), which delivered a capacity of 345 mAh g^−1^ at 100 mA g^−1^ with a PCE of 0.2% under 1 sun. Nevertheless, the less‐than‐ideal band edge matching of conduction band (CB) and valence band (VB) values between ZnO and MoS_2_ (△*E*_CB_ = 0.1 eV, △*E*_VB_ = 1.5 eV) also negatively affects the charge separation on photocathodes of PRZIBs. Hence, the unsatisfactory structural design and the band edge mismatch of photosensitive and electronic transport species lead to sluggish reaction kinetics and inefficient charge separation on photocathodes, thus limiting the overall efficiency of PRZIBs. Therefore, designing an optimal electrode structure with matching material energy levels and band edges for photocathodes remains a key challenge to achieving a higher PCE of PRZIBs.^[^ ^41^ ^]^

Inspired by the interlaced structure of sunflower petals with an extreme ability in utilization of solar energy,^[^ ^42^, ^43^ ^]^ we have designed a sunflower‐shaped, 3D hierarchical, and self‐supporting light‐trapping nano‐structure for MoS_2_/SnO_2_‐based photocathode in PRZIBs, which was enabled by the hierarchical embellishment of MoS_2_ nanosheets and SnO_2_ quantum dots (QDs) on carbon cloth (MoS_2_/SnO_2_ QDs@CC). In particular, the 3D‐hierarchical and self‐supporting structure design guarantees multiple electrochemical activity sites of photocathodes, and the appropriate band edge matching of CB and VB positions between MoS_2_ and SnO_2_ (△*E*_CB_ = 0.1 eV, △*E*_VB_ = 1.7 eV) promotes effective separation of photogenerated electron‐hole pairs under illumination, undoubtedly boosting reaction kinetics and energy density of PRZIBs. Furthermore, the highly photosensitive MoS_2_ with sunflower‐shaped nano‐structure facilitates light absorption and trapping, while the SnO_2_ QDs layer shortens the diffusion path and accelerates electron transport.^[^ ^44^, ^45^, ^46^, ^47^ ^]^ As expected, the PRZIB assembled with MoS_2_/SnO_2_ QDs@CC photocathode delivers a desirable specific capacity of 366 mAh g^−1^ and an ultra‐high PCE of 2.7%, which outperforms that of reported PRZIBs employing 2D‐planar photocathodes. For practical application, an integrated battery system consisting of four series‐connected quasi‐solid‐state PRZIBs is successfully assembled in a wearable device of smartwatches and demonstrates outstanding operational stability and mechanical flexibility, confirming the feasibility of integrating MoS_2_/SnO_2_ QDs@CC PRZIBs in wearable electronic devices.

## 2 Results and Discussion

### 2.1 Synthesis and Characterization of MoS_2_/SnO_2_ QDs@CC

**Figure 1:**
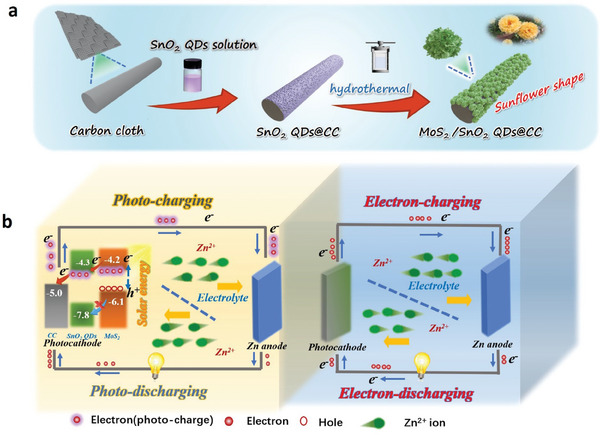
Synthesis and photo‐charging mechanism of MoS_2_/SnO_2_ QDs@CC. a) Synthetic route of MoS_2_/SnO_2_ QDs@CC; b) Energy band matching diagrams of MoS_2_/SnO_2_ QDs @CC and working mechanism of PRZIBs under illuminated and dark conditions, respectively.

The two‐step synthetic route of the MoS_2_/SnO_2_ QDs@CC photocathode is shown schematically in **Figure** **1**a. First, the transparent and yellow colloidal solution of SnO_2_ QDs is synthesized by a facile solution method, in which the Tyndall effect can be observed (Figure S1a, Supporting Information). By immersing CC substrate in SnO_2_ QDs solution, a SnO_2_ QDs layer can be formed on CC (SnO_2_ QDs@CC) through physical adsorption. Subsequently, the MoS_2_/SnO_2_ QDs@CC is obtained by hydrothermal processing of SnO_2_ QDs@CC, during which MoS_2_ nanosheets are in situ interlaced grown on SnO_2_ QDs@CC owing to the electronegative effect of SnO_2_ and the radial lattice growth tendency of MoS_2_. The detailed synthesis processes of SnO_2_ QDs solution and MoS_2_/SnO_2_ QDs@CC are shown in the experimental section.

The detailed charging/discharging mechanism of MoS_2_/SnO_2_ QDs @CC photocathode under illuminated and dark conditions is shown in Figure 1b. During the charging/discharging process, Zn^2+^ is reversibly deintercalated/intercalated from/to MoS_2_/SnO_2_ QDs @CC, and its electrochemical behavior is no different from that of a normal Zn ion battery. When the charging process is excited by illumination without an external driving force, the electrons generated by the electron‐hole pair separation in MoS_2_ can easily pass through the SnO_2_ QDs layer and quickly transfer to the CC substrate, owing to the close CB values of MoS_2_ and SnO_2_ (Figure S2, Supporting Information). Then, the transferred photo‐excited electrons reach the Zn anode via the external circuit to complete the reduction of Zn metal. However, due to the large difference between the two VB values, the photo‐excited holes in MoS_2_ are blocked and drive the deintercalation of Zn^2+^ from MoS_2_. For the photo‐discharging process, the built‐in electric field formed by photogenerated electron‐hole pairs hinders the transfer of electrons from the external circuit to the photocathode, thereby slowing down the decline of photocathode potential and enhancing the discharge capability compared to that in the dark.

**Figure 2:**
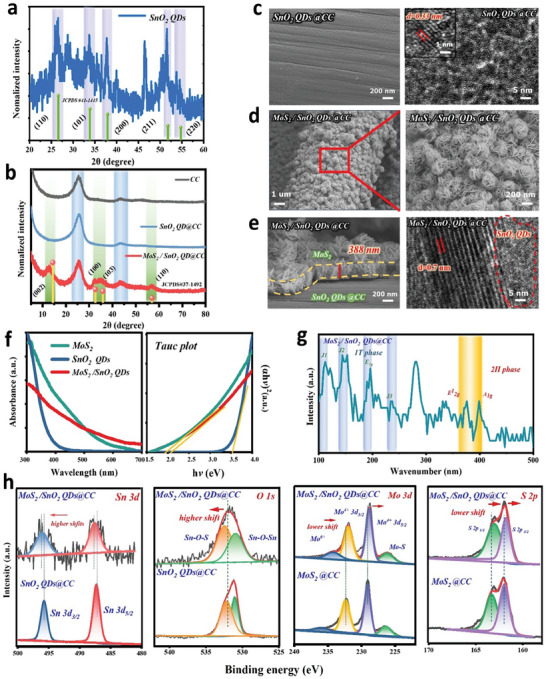
Morphology and structure characteristics of MoS_2_/SnO_2_ QDs@CC. a,b) XRD patterns; c–e) SEM, TEM, and HRTEM images; f) UV–vis absorption spectrum and Tauc plot; g) Raman spectra; h) XPS spectra of SnO_2_ QDs@CC, MoS_2_@CC and MoS_2_/SnO_2_ QDs@CC.

A series of physical characterizations of the MoS_2_/SnO_2_ QDs@CC photocathode were carried out as follows. The X‐ray diffraction (XRD) pattern (**Figure** **2**a) shows the diffraction peaks of as‐prepared SnO_2_ QDs, which is consistent with the SnO_2_ phase from JCPDS^#^41‐1445. As confirmed by the transmission electron microscope (TEM) image (Figure S1b,c, Supporting Information), the particle size of SnO_2_ QDs is 0.98 ± 0.19 nm. After immersing the CC substrate (SEM image in Figure S3, Supporting Information) to SnO_2_ QDs solution, a uniform and dense SnO_2_ layer is distributed on the CC substrate (Figure 2c left). The corresponding high‐resolution TEM (HRTEM) (Figure 2c right) identifies the d‐spacing value of 0.33 nm, assigned to the (110) crystal plane of the SnO_2_ species. Figure 2b demonstrates that the XRD patterns of the MoS_2_/SnO_2_ QDs@CC, SnO_2_ QDs@CC, and CC, where the diffraction peaks of MoS_2_ (JCPDS^#^37‐1492) can be clearly observed on MoS_2_/SnO_2_@CC, indicating that the MoS_2_ was successfully modified in situ on SnO_2_ QDs layer. It's worth noting that the SnO_2_ characteristic peak cannot be observed on SnO_2_ QDs@CC, which is attributed to the low content of SnO_2_ QDs and the stronger characteristic peaks of CC.

Figure 2d illustrates the microstructure of in situ modified MoS_2_, which is vertically staggered on the SnO_2_ QDs layer (left) and possesses a nanoscale sunflower‐like morphology (right). Furthermore, the cross‐section of 3D‐hierarchical MoS_2_/SnO_2_ QDs@CC is depicted in Figure 2e (left), clearly showing a 3D sunflower‐like MoS_2_ layer with a thickness of 388 nm. The corresponding HRTEM image (Figure 2e right) presents the crystal planes of (002) from MoS_2_ and (110) from SnO_2_ at the phase junction, implying a strong contact between MoS_2_ and SnO_2_, which could effectively promote carrier transport efficiency.

The ultraviolet‐visible (UV–vis) spectrum in Figure 2f (left) confirms the broader photo‐response range of MoS_2_/SnO_2_ QDs@CC compared to MoS_2_ and SnO_2_ alone. According to (α*hv*)^1/m^ = B(*hv*‐*E_g_*) equation, the Tauc plots are further provided in Figure 2f (right). The band gap (*E_g_*) values of the three samples are 2.1 eV for MoS_2_/SnO_2_ QDs, 3.5 eV for SnO_2_, and 1.9 eV for MoS_2_, respectively. Figure 2g exhibits the Raman spectrum of MoS_2_/SnO_2_@CC in the range of 100–500 nm, in which the peaks located at 382 cm^−1^ (in‐plane E^1^_2 g_ vibration) and 401 cm^−1^ (vertical plane A_1g_ vibration) are related to the 2H phase of MoS_2_. The difference (*△k*) between A_1g_ and E^1^_2 g_ reflects the number of MoS_2_ layers. The *△k* value of MoS_2_ for MoS_2_/SnO_2_ QDs@CC is 23 cm^−1^ (equivalent to 3 layers), which is smaller than the expected value of bulk MoS_2_ (25.7 cm^−1^).^[^ ^48^ ^]^ The smaller number of layers indicates a greater spacing of MoS_2_, which suggests that MoS_2_ has undergone lattice distortion during the synthesis process. And the obvious peaks at 143, 192, 224, and 279 cm^−1^ are associated with its 1T phase, according to previous reports, the 1T phase of MoS_2_ is electrochemically active and hydrophilic, while its 2H phase exhibits thermodynamically stability and semiconducting properties. Therefore, a reasonable mixing of 1T and 2H phases of MoS_2_ is a favorable strategy to improve the storage capacity and stability of Zn^2+^ in MoS_2_‐based electrodes.^[^ ^49^, ^50^ ^]^

The elemental composition and valence state of the MoS_2_/SnO_2_ QDs@CC photocathode was determined via X‐ray photoelectron spectroscopy (XPS). The obtained full spectrum is shown in Figure S4 (Supporting Information), indicating that the MoS_2_/SnO_2_ QDs@CC comprises Mo, Sn, S, O, and C without other impurities. At the same time, energy dispersive X‐ray spectroscopy (EDS) was also performed on MoS_2_/SnO_2_ QDs@CC, and the uniform dispersion of Mo, S, Sn, and O elements can be observed (Figure S5, Supporting Information). In addition, the XPS spectra of Sn 3d, O 1s, Mo 3d, and S 2p of SnO_2_ QDs@CC, MoS_2_@CC, and MoS_2_/SnO_2_ QDs@CC were further analyzed as demonstrated in Figure 2h. Notably, the binding energy of Sn 3d_3/2_ and Sn 3d_5/2_ peaks in Sn 3d and Sn─O─S peak in O 1s spectra of MoS_2_/SnO_2_ QDs@CC shifts to a higher value in contrast to that of SnO_2_ QDs@CC, whereas the binding energy of Mo^4+^ 3d_3/2_ and Mo^4+^ 3d_5/2_ peaks in Mo 3d spectra and S 2p_1/2_ and S 2p_5/2_ peaks in S 2p spectra in MoS_2_/SnO_2_ QDs@CC shifts to a lower value compared with MoS_2_@CC. These shifts imply that electrons are transferred from MoS_2_ to SnO_2_ in MoS_2_/SnO_2_ QDs heterojunction, indicating a strong electronic interaction between SnO_2_ and MoS_2_ in MoS_2_/SnO_2_ QDs@CC.

Finally, the electrochemical active surface area of MoS_2_/SnO_2_ QDs@CC photocathode (Figure S6a, Supporting Information) was evaluated by the double‐layer capacitance (C_dl_), affording 224.5 mF cm^−2^ (Figure S6c, Supporting Information), which is considerably higher than that of the CC alone (12.6 mF cm^−2^) in Figure S6b (Supporting Information). The ultra‐high double‐layer capacitance indicates that MoS_2_/SnO_2_ QDs@CC can provide abundant active sites for light harvesting and Zn^2+^ storage, which benefits from its sunflower‐shaped light‐trapping nano‐structure with 3D‐hierarchical and self‐supporting features.^[^ ^51^ ^]^ Furthermore, MoS_2_/SnO_2_ QDs@CC presents a smaller water contact angle of 79.78° than that of bare CC (133.38°) in Figure S7 (Supporting Information), suggesting that a favorable diffusion kinetics can be obtained at the interface between material and electrolyte.

### 2.2 Photoelectric Response Properties of Photoelectrodes

**Figure 3:**
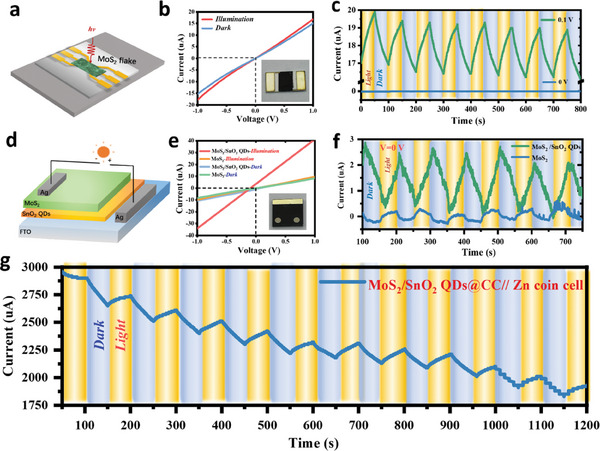
Photoelectric response properties of photoelectrodes. a) Schematic illustration of MoS_2_ planer PD, b) its *I–V* curves, and c) current‐time (*I–t*) curves recorded under alternating dark and illumination conditions at a bias voltage of 0.1 and 0 V; d) Schematic illustration of MoS_2_/SnO_2_ QDs stacked PD, e) *I–V* curves of different stacked PDs and f) their *I–t* curves recorded under alternating dark and illumination conditions; g) The *I–t* curve of MoS_2_/SnO_2_ QDs@CC // Zn coin cell.

To further investigate the photoelectric response properties in MoS_2_/SnO_2_ heterojunction, the planar and stacked photodetectors (PDs) based on heterojunction were fabricated and tested under a light intensity of 1 sun (100 mW cm^−2^). As illustrated in **Figure** **3**a, the planar PDs were fabricated by patterning Au/Cr onto interdigital electrodes (IDEs) and then drop‐casting slurry with MoS_2_ nanosheets on the IDEs. Figure 3b shows the current‐voltage (*I–V*) curves of the planar PD under dark and illumination conditions, where the photosensitivity of MoS_2_ nanosheets can be confirmed by a slight increase of photocurrent under illumination, the inset shows the photograph of the MoS_2_ photodetector. The current‐time (*I–t*) curves (Figure 3c) demonstrate a photoelectric response effect at a bias voltage of 0.1 V but not at 0 V, recorded under alternating dark and illuminated conditions. This finding suggests that MoS_2_ planer PD requires an external driving force (bias voltage) for separating photo‐excited electrons and holes under illumination. We further fabricated a MoS_2_/SnO_2_ QDs heterojunction stacked PD device, as depicted in Figure 3d. The well‐designed stacked PD is based on a hierarchical stacking of fluorine‐doped tin oxide (FTO) electrode, MoS_2_/SnO_2_ QDs heterojunction, and top contact silver (Ag) electrode. Obviously, the MoS_2_/SnO_2_ QDs stacked PD shows much higher photosensitivity (Figure 3e) than that of pure MoS_2_ stacked PD, implying that the proposed MoS_2_/SnO_2_ QDs heterojunction facilitates the separation of photo‐excited charges. Figure 3f further confirmed the *I–t* curves of pure MoS_2_ and MoS_2_/SnO_2_ QDs stacked PDs without bias voltage, recorded under alternating dark and illuminated conditions. The photoelectric response of MoS_2_/SnO_2_ QDs stacked PDs is more noticeable than that of the MoS_2_ one, which emphasizes the necessity of MoS_2_/SnO_2_ heterojunction for highly efficient photovoltaic processes. Further, to demonstrate the suitability of MoS_2_/SnO_2_ QDs@CC photocathode for PRZIBs, a MoS_2_/SnO_2_ QDs@CC//Zn coin cell was assembled, and its chronopotentiometric response at 0 V was also recorded under the same condition. Figure 3g displays that a desired sensitive photoelectric responsibility is maintained for MoS_2_/SnO_2_ QDs@CC Zn coin cell, which outperforms other reported 2D‐planar photocathodes in different photo‐rechargeable batteries systems (Table S1, Supporting Information), showing the superiority of 3D‐hierarchical and self‐supporting design of MoS_2_/SnO_2_ QDs@CC photocathodes for PRZIBs.

Particularly, the outstanding photoelectric response performance of MoS_2_/SnO_2_ QDs@CC is also attributed to its light absorption and capture properties. As confirmed by Figure S8 (Supporting Information), the absorbance of MoS_2_/SnO_2_ QDs@CC (86%) is higher than that of CC (71%). In addition, Figure S9 (Supporting Information) reveals that a higher apparent temperature is detected on MoS_2_/SnO_2_ QDs@CC (61.2 °C) compared with CC (54.9 °C) after light irradiation for over 2 h. These results strongly validate that the sunflower‐shaped light‐trapping material structure of MoS_2_/SnO_2_ QDs@CC favors the absorption and capture of light. In order to understand the influence of the photo‐thermal effect, the electrochemical performance of the battery without an optical window under illuminated and dark conditions was compared. The overall electrochemical performance (Figure S10, Supporting Information) is essentially the same, except for a slight increase in capacity when affected by light. This result suggests that the photo‐thermal effect has a minimal impact on the improvement of the electrochemical performance of the MoS_2_/SnO_2_ QDs @CC PRZIB.

### 2.3 Electrochemical Performance of MoS_2_/SnO_2_ QDs@CC PRZIBs

**Figure 4:**
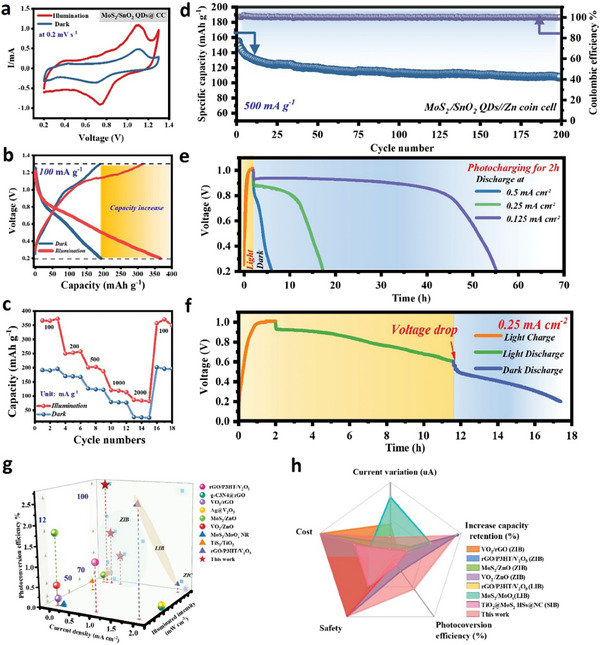
Electrochemical performance of MoS_2_/SnO_2_ QDs@CC PRZIBs. a) *CV* curves, b) galvanostatic charge/discharge curves c) and rate performance of PRZIBs under dark and illumination conditions; d) Cycling performance of PRZIB at a current density of 500 mA g^−1^ in dark; e) Voltage‐time (*V–t*) curves of PRZIBs with photo‐charging for 2 h and discharging at different specific currents in dark; f) *V–t* curves of PRZIB with photo‐charging for 2 h and discharging at 0.25 mA cm^−2^ current density under different conditions; g) Comparison of photovoltaic conversion efficiency of different photo‐rechargeable cell systems; h) Comparison of overall performance.

The electrochemical performance of the PRZIB assembled with MoS_2_/SnO_2_ QDs@CC photocathode was evaluated by cyclic voltammetry (CV) and galvanostatic charge/discharge (GCD) techniques in dark and illumination. As shown in **Figure** **4**a, the CV curves recorded at 0.2 mV s^−1^ under dark and illuminated conditions exhibit the same redox (located at 0.7 V) and oxide (located at 1.1 V) peak positions, which correspond to the intercalation and de‐intercalation reactions of Zn^2+^ with MoS_2_, respectively.^[^ ^52^, ^53^ ^]^ Notably, the current intensity of the CV curve recorded under illumination (Figure S11b, Supporting Information) is higher than that of in the dark (Figure S11a, Supporting Information), which is determined by the kinetic of Zn^2+^ diffusion. To further understand the kinetic processes of MoS_2_/SnO_2_ QDs@CC during the Zn^2+^ intercalation/deintercalation process, the Zn^2+^ diffusion coefficient (*D*) under dark and illuminated conditions are detected and calculated by eq.S1 (Figure S11c, Supporting Information). As a result, the *D* for Zn^2+^ intercalation and deintercalation are 3.92×10^−11^ cm^2^ s^−1^ and 7.20×10^−11^ cm^2^ s^−1^ under illumination, respectively, which are significantly 10 times higher than those in dark (7.34×10^−12^ cm^2^ s^−1^ and 4.83×10^−12^ cm^2^ s^−1^). Compared to related work,^[^ ^54^ ^]^ the *D* of Zn^2+^ diffusion ranges from 1×10^−14^ to 1×10^−12^. It highlights the favorable intercalation/deintercalation kinetics of MoS_2_/SnO_2_ QDs@CC. It suggests that faster kinetics of Zn^2+^ intercalation/deintercalation can be obtained on MoS_2_/SnO_2_ QDs@CC under illumination.

By linear fitting the plot of log(***i***) versus log(***v***) (Figure S12a,b, Supporting Information), the calculated *b* values for the cathodic/anodic peaks are ≈0.86/0.83 in dark and ≈0.83/0.77 in illuminated condition, indicating that the charge storage of MoS_2_/SnO_2_ QDs@CC photocathode is mainly controlled by pseudo‐capacitance behavior. As shown in Figure S12c (Supporting Information), we performed eq.S2 calculations of the anodic peak current for different scans under dark and illuminated conditions. These results reveal the proportion of contributions from capacitive and diffusion‐controlled processes in charge storage, suggesting the diffusion‐controlled contribution would be greater in the presence of light. Otherwise, as illustrated in Figure S12d (Supporting Information), the capacitive contribution derived from eq.S3 is ≈63.8% in light at 1 mV s^−1^ compared to ≈73.5% in dark, which further confirms that photo‐generated charges enhance the diffusive contribution of MoS_2_/SnO_2_ QDs@CC photocathode in charge storage.

GCD test was carried out under a current density of 100 mA g^−1^, as shown in Figure 4b. The results of GCD profiles are consistent with the CV curves, in which a gently inclined discharge/charge platform starting from 0.75/1.1 V correlates to the intercalation/deintercalation of Zn^2+^ in MoS_2_. However, it is noteworthy that the PRZIB tested under illumination delivers a desirable discharge/charge specific capacity of 366/320 mAh g^−1^ and remarkably higher than that of in dark (190/189 mAh g^−1^), which is attributed to the influence of photo‐assisted charging on MoS_2_/SnO_2_ QDs@CC photocathode during discharge/charge process. For comparison, the delivered specific capacity of photocathodes in different photo‐rechargeable battery systems were listed in Table S2 (Supporting Information). Under a light intensity of 100 mW cm^−2^, the increased capacity of MoS_2_/SnO_2_ QDs@CC in PRZIB is up to 92.6%, which outperforms those reported photo‐rechargeable battery systems. Figure 4c shows the rate capacity performance of PRZIBs under the current density range of 100 to 2000 mA g^−1^. The PRZIBs deliver higher specific capacities under illumination compared with those in the dark, and the voltage gap generated under illumination is lower than that of in the dark (Figure S13, Supporting Information), indicating the superior rate capability and lower polarization potential of PRZIBs in response to light stimulation. It is worth noting that the capacity of the battery tested under light is higher at low specific currents. This is because at high current densities, the photo‐extracted Zn^2+^ from MoS_2_ would be enriched at the electrode/electrolyte interface, which leads to the phenomenon of concentration polarization and reduces the efficiency of current conduction. This is because the charge transfer resistance (*R_ct_*) of PRZIB under illumination is ≈21 Ω, far smaller than that of in the dark (77 Ω), further manifesting that the photoexcited carriers facilitate the intercalation/deintercalation kinetics of Zn^2+^ under illumination (Figure S14, Supporting Information).

The PRZIB also exhibits an excellent cyclic performance at a current density of 500 mA g^−1^ in dark conditions, as shown in Figure 4d. The PRZIB exhibits a slow decay in specific capacity and maintains 107 mA g^−1^ after 200 cycles. Also, the coulombic efficiency maintains a high level of 99.57%, which highlights the reversibility of MoS_2_/SnO_2_ QDs@CC. To understand the influence of photo‐charging on PRZIBs, some special charging and discharging procedures were carried out. Figure 4e depicts the voltage‐time (*V–t*) curves of PRZIBs charged under illumination only (without any external electric power supplied) and subsequently discharged in the dark at different current densities. After photo‐charging for 2 h, the open‐circuit voltage of PRZIBs rises from 0.2 to 0.962 V. And accompanied by continuous discharge to 0.2 V at current densities of 0.125, 0.25, and 0.5 mA cm^−2^, the discharge durations of PRZIBs are 53.5, 15.3 and 4.1 h, respectively. Based on the above discharge results, we could quantify the charge stored on PRZIBs during the photo‐charging process, and the corresponding PCE is calculated by the following relation:

(1) $$PCE\left(\%\right)=E_{out}/E_{in}\times 100\%=E_{1}\times I\times t_{1}/P_{in}\times t_{2}\times A$$

where *E*_1,_ *I, P*_in,_ *A*, *t*_1,_ and *t*_2_ represent the voltage drop during the discharge process, the discharge current density, the light intensity, the illuminated area, and the discharge, and the illuminated time, respectively. As a result, the PCE of PRZIBs at current densities of 0.125, 0.25, and 0.5 mA cm^−2^ are 2.7%, 1.4%, and 0.79%, respectively, which is higher than other photocathodes in different battery systems as reported (Table S3, Supporting Information). Furthermore, as illustrated in Figure 4f, after photo‐charging the PRZIB for 2 h, the voltage of PRZIB slowly drops from 0.85 to 0.6 V at a constant current density of 0.25 mA cm^−2^ owing to the simultaneous photo‐charging effect. Obviously, when the light source is suddenly switched off, the discharge rate of PRZIB becomes fast and a turning point occurs.

Figure 4g shows a comparison of the photoelectric conversion efficiency of different PRZIB systems. In comparison, the photoelectric conversion efficiency of this device is significantly higher than that of the previously reported PRZIB, PRZIC, and PRLIB systems. In addition, as shown in Figure 4h, we also conducted a comprehensive evaluation of the reported photo‐rechargeable battery systems in terms of cost, safety, current increase, and capacity growth rate, and the results indicate that the PRZIB system with MoS_2_/SnO_2_ QDs@CC as the photocathode is a high‐performance, high‐efficiency, low‐cost, and high‐capacity energy storage device.

### 2.4 Intrinsic Properties of MoS_2_/SnO_2_ QDs@CC and Structural Evolution during Charging/Discharging Process

**Figure 5:**
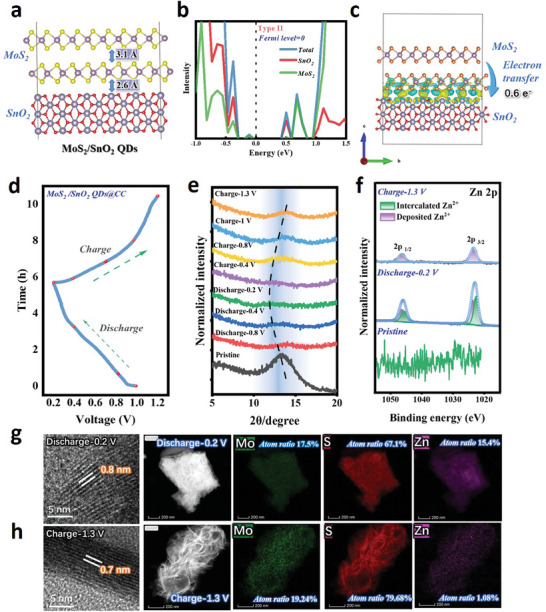
Intrinsic properties of MoS_2_/SnO_2_ QDs@CC and structural evolution during charging and discharging process. DFT‐optimized a) structural models, b) PDOS and c) differential charge density distribution of MoS_2_/SnO_2_ heterojunction; d) Galvanostatic 1st discharge–charge cycle curve of MoS_2_/SnO_2_ QDs@CC PRZIB; e) Ex situ XRD patterns and f) ex situ XPS spectra of Zn element for MoS_2_/SnO_2_ QDs@CC at selected voltages; HRTEM and mapping elements images of MoS_2_/SnO_2_ QDs@CC g) discharged to 0.2 V and h) charged to 1.3 V.

To gain an in‐depth insight into the intrinsic properties of MoS_2_/SnO_2_ QDs@CC photocathode, we investigated the structural characters, Zn^2+^ adsorption, and migration energy by first‐principles theory. First, the structural characters of MoS_2_/SnO_2_ heterojunction were evaluated using the density functional theory (DFT) calculations.^[^ ^55^, ^56^ ^]^ **Figure** **5**a and Figure S15a (Supporting Information) show the DFT‐optimized structures of isolated MoS_2_ bilayer, isolated SnO_2_ and MoS_2_/SnO_2_ heterojunction. The optimized interlayer distance between MoS_2_ and SnO_2_ is 2.6 Å, and the corresponding calculated binding energy is −6.6 eV. The short interlayer distance and large binding energy between MoS_2_ and SnO_2_ indicate a strong interaction at the interface. Figure 5b shows the projected density of states (PDOS) results, suggesting that the energy levels of MoS_2_/SnO_2_ heterojunction are arranged in accordance with type‐II band alignment, in which the VB maximum and CB minimum of the heterojunction originate from MoS_2_ and SnO_2_, respectively. Figure 5c shows charge redistribution at the MoS_2_/SnO_2_ interface, where the yellow and cyan regions represent accumulation and depletion of charge, respectively. The charge depletion on MoS_2_ and the charge accumulation on SnO_2_ confirm the formation of the built‐in electric field at the interface, which can promote rapid electron transfer between MoS_2_ and SnO_2_. Based on Bader charge calculations of MoS_2_/SnO_2_ heterojunction, the electrons on MoS_2_ are transferred to SnO_2_ with an amount of 0.6 e^−^, which is consistent with the XPS results of Figure 2h. Next, the Zn^2+^ adsorption and migration energy of MoS_2_/SnO_2_ heterojunction were also obtained by DFT methods. As shown in Figure S15b (Supporting Information), the adsorption and migration energy of Zn^2+^ on MoS_2_/SnO_2_ heterojunction are −0.17 and 0.37 eV, respectively, close to those of isolated MoS_2_ bilayer (−0.15 and 0.32 eV), which implies that the introduce of SnO_2_ has no negative effect on the Zn^2+^ intercalation/deintercalation kinetics of MoS_2_.

Through ex situ X‐ray technique and structural characterization, more direct evidences can be obtained to identify the storage mechanism of Zn^2+^ in MoS_2_/SnO_2_ QDs@CC photocathodes for PRZIBs. The MoS_2_/SnO_2_ QDs@CC photocathode in PRZIBs under selected discharge and charge states (Figure 5d) were first investigated by ex situ XRD (Figure 5e). The resulting XRD profiles suggest that the diffraction peak from the (002) crystal plane of MoS_2_ gradually shifts to a smaller 2θ angle during the discharge process, indicating that the interlayer spacing of MoS_2_ increases along with the intercalation of Zn^2+^. However, in subsequent charging process, the diffraction peak from the (002) crystal plane almost returns to its original 2θ angle, which strongly confirms the high reversibility of Zn^2+^ intercalation/deintercalation on MoS_2_/SnO_2_ QDs@CC photocathode. Further, ex situ XPS characterization was conducted to track the compositional changes of MoS_2_/SnO_2_ QDs@CC under a fully charged and discharged state. The Zn 2p spectrum (the bottom of Figure 5f) confirms the absence of Zn species in the pristine MoS_2_/SnO_2_ QDs@CC. When the PRZIB is fully discharged to 0.2 V, two pairs of deconvoluted Zn peaks appear in the Zn 2p spectrum (the middle of Figure 5f), which are assigned to the Zn^2+^ intercalation into MoS_2_ and the electrolyte decomposition product Zn (OH)_2_ deposited on MoS_2_/SnO_2_ QDs@CC, respectively. After the full charging of PRZIB to 1.3 V, only the characteristic peak of Zn (OH)_2_ can be observed in the Zn 2p spectrum (the top of Figure 5f), implying the Zn^2+^ deintercalation from MoS_2_. Figure 5g,h shows the HRTEM images of MoS_2_/SnO_2_ QDs@CC at charged and discharged state, visually describing the change of lattice spacing during the Zn^2+^ intercalation/deintercalation process, which is consistent with the ex situ XRD results (Figure 5e). Finally, the EDS spectra of Mo, S, and Zn elements and their relative ratio reveal the uniform intercalation/deintercalation of Zn^2+^ in/from MoS_2_/SnO_2_ QDs@CC heterojunction during the charge and discharge process. And the corresponding element analysis (Figure S16, Supporting Information) shows that the Zn element content (15.4 *at*%, *at*, atomic ratio) in MoS_2_/SnO_2_ QDs@CC heterojunction at fully discharged state is far higher than that of a fully charged state (1.08 *at*%), again confirming the electrochemical reversibility of MoS_2_/SnO_2_ QDs@CC during Zn^2+^ intercalation/deintercalation.

### 2.5 Applications of Flexible Quasi‐Solid‐State PRZIBs for Wearable Electronic Devices

**Figure 6:**
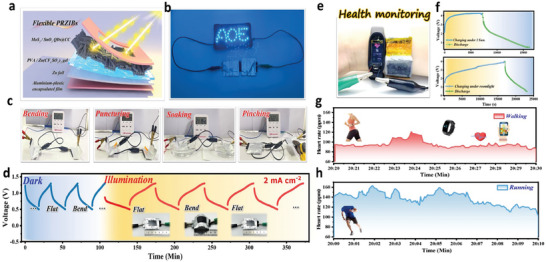
Applications of flexible QSSPZs for wearable electronic devices. a) Schematic diagram of the flexible QSSPZ; b) Optical image of a LED signboard with “AOE” shape powered by two series‐connected QSSPZs; c) A series safety tests of the QSSPZs under different conditions, including bending, puncturing, soaking and pinching; d) *V–t* curves of a flexible QSSPZs tested in alternating flat and bend states under dark and illuminated conditions; e) Optical image of a smartwatch powered by four series‐connected QSSPZs, f) and their *V–t* curves charged under sunlight and room light conditions and then discharged in dark; g) Dynamic information obtained from the integrated smartwatch during walking and h) running processes.

For applications, we have further developed a flexible quasi‐solid‐state PRZIB (QSSPZs) comprised of a MoS_2_/SnO_2_ QDs@CC photocathode and a Zn foil anode sandwiched with poly(vinyl alcohol) (PVA)/Zn (CF_3_SO_3_)_2_ gel electrolyte (**Figure** **6**a), which can meet the integrated and wearable requirements of battery systems.^[^ ^19^, ^57^, ^58^, ^59^, ^60^, ^61^ ^]^ As shown in Figure S17 (Supporting Information), this QSSPZ can generate an open circuit potential of 1.226 V, and deliver a high capacity of 1885/1318 mAh under illumination/dark conditions. Interestingly, two QSSPZs connected in series can successfully light 30 blue LEDs (2.2 V) with an “AOE” shape (Figure 6b). Furthermore, even under a variety of extreme conditions including bending, puncturing, soaking, and pinching (Figure 6c), the two series‐connected QSSPZs can still work perfectly, demonstrating the impressive safety and practicality in the integrated devices. To further evaluate the operational stability and mechanical flexibility of the assembled QSSPZs, it was carefully tested via GCD at a constant current of 2 mA cm^−2^. Under the respective conditions of dark and illumination (Figure 6d), the charging and discharging voltage profiles are almost the same in both flat and bent states, which confirms the excellent operational stability and mechanical flexibility of QSSPZs.

To practically apply the QSSPZs to wearable electronic devices,^[^ ^62^, ^63^, ^64^, ^65^ ^]^ we designed a flexible battery system consisting of four series‐connected QSSPZs as a wearable wristband for smartwatches (Figure 6e). As illustrated in Figure 6f, this battery system is charged up to 4.2 V for 2 h under 1 sun and discharged for 2.5 h in the dark at a current density of 0.5 mA cm^−2^, which also can work perfectly even under room light (charged up to 4.2 V for 4.5 h and discharged for 1.7 h at 0.5 mA cm^−2^), demonstrating the great potential for all‐day operation. In practice, the integrated smartwatch can continuously monitor the heart rate date of exercisers while running and walking outdoors (Figure 6g,h). Thanks to the superior operational reliability of QSSPZs in wristbands (Figure S18, Supporting Information), the smart watch can record exercise data in real time and send it to a mobile phone for monitoring and analysis. The above results demonstrate the feasibility of integrating MoS_2_/SnO_2_ QDs@CC PRZIBs into wearable and portable electronics, which will drive great advances in the next generation of flexible energy storage devices.

## 3 Conclusion

In summary, we have designed and fabricated a sunflower‐shaped light‐trapping nano‐structure with 3D‐hierarchical and self‐supporting features for MoS_2_/SnO_2_‐based photocathode (MoS_2_/SnO_2_ QDs@CC). In particular, the highly photosensitive sunflower‐shaped MoS_2_ nano‐structure facilitates light absorption and trapping, while simultaneously providing abundant electrochemical sites for Zn^2+^ intercalation/deintercalation. The unique design of the SnO_2_ QDs layer can shorten the diffusion path, resulting in faster electron transport. In addition, the interfacial contact between MoS_2_/SnO_2_ heterojunction creates an internal electric field, which further improves electron‐hole separation and transfers photo‐carries under illumination. Benefiting from the well‐designed MoS_2_/SnO_2_ QDs@CC photocathode, its PRZIB discharged at 100 mA g^−1^ delivers a desirable specific capacity of 366 mAh g^−1^ under a light intensity of 100 mW cm^−2^, which is almost twice the specific capacity in dark (190 mAh g^−1^). For practical application, a flexible QSSPZ assembled with the MoS_2_/SnO_2_ QDs@CC photocathode exhibits high open circuit potential (1.226 V), high capacity (1883 mAh), excellent operational stability, as well as outstanding mechanical flexibility. More impressively, a wearable smart watch integrated battery system consisting of four series‐connected QSSPZs is developed, demonstrating its successful application to realistic wearable electronic devices. The remarkable progress in PRZIB photocathodes holds great promise for the applications of next‐generation flexible energy storage devices.

## Conflict of Interest

The authors declare no conflict of interest.

## Supporting information

Supporting Information.

## Data Availability

Research data are not shared.
