# Supplementary material for: 3D Hierarchical Sunflower‐Shaped MoS2/SnO2 Photocathodes for Photo‐Rechargeable Zinc Ion Batteries
Source: Adv Sci (Weinh). 2024 Mar 19;11(21):2309555. doi: 10.1002/advs.202309555 (PMC11151025; doi:10.1002/advs.202309555)
Supplement: Supplementary file 1 — Supporting Information. [file ADVS-11-2309555-s001.pdf]

ADVANCED SCIENCE
Open Access

# Supporting Information

for *Adv. Sci.*, DOI 10.1002/advs.202309555

3D Hierarchical Sunflower-Shaped $MoS_2/SnO_2$ Photocathodes for Photo-Rechargeable Zinc Ion Batteries

*Xinyang Wen, Yaotang Zhong*, Shuai Chen, Zhengchi Yang, Pengyu Dong, Yuqi Wang, Linghai Zhang*, Zhen Wang, Yue Jiang, Guofu Zhou, Junming Liu and Jinwei Gao**

***Supporting information***

# 3D Hierarchical Sunflower-Shaped $MoS_2/SnO_2$ Photocathodes for Photo-Rechargeable Zinc Ion Batteries

Xinyang Wen[1&], Yaotang Zhong[1]*, Shuai Chen[2], Zhengchi Yang[1], Pengyu Dong[1], Yuqi Wang[1], Linghai Zhang[3]*, Zhen Wang[1], Yue Jiang[1], Guofu Zhou[4], Junming Liu[5], Jinwei Gao[1,6]*

1. Institute for Advanced Materials and Guangdong Provincial Key Laboratory of Quantum Engineering and Quantum Materials, South China Academy of Advanced Optoelectronics, South China Normal University, Guangzhou 510006, China
2. School of Chemistry, South China Normal University, Guangzhou 510006, China
3. School of Flexible Electronics (Future Technologies), Nanjing Tech University, Nanjing, 211816, China
4. Guangdong Provincial Key Laboratory of Optical Information Materials and Technology & Institute of Electronic Paper Displays, South China Academy of Advanced Optoelectronics, South China Normal University, Guangzhou 510006, China
5. Laboratory of Solid State Microstructures, Nanjing University, Nanjing 210093, China
6. Centre for Advanced Optoelectronics, School of Physics and Electronic Information, Gannan Normal University, Ganzhou, Jiangxi 341000, China

E-mail: gaojinwei@gnnu.edu.cn (J. W. G.); zhongyaotang@scnu.edu.cn (Y. T. Z); iam.lzhang@njtech.edu.cn (L.Z.)

## Content of the supporting information:

## 1. Experimental Section

***1.1*** Material preparation

***1.2*** Material characterization

***1.3*** Manufacturing and electrochemical testing of photodetectors

***1.4*** Assembly of PRZIBs

***1.5*** Assembly of flexible pouch cells

***1.6*** Electrochemical test of PRZIBs

***1.7*** Wearable demonstration

***1.8*** DFT calculation

## 2. Supplementary Figures and Tables

**Figure S1.** Digital graph of a laser beam irradiated $SnO_2$ QDs solution (a); The HRTEM image (b) and particle size distribution (c) of $SnO_2$ QDs solution.

**Figure S2.** The UPS curves of $MoS_2$@CC and $SnO_2$ QDs@CC.

**Figure S3.** SEM image of carbon cloth.

**Figure S4.** Full XPS spectra of $MoS_2/SnO_2$ QDs@CC.

**Figure S5.** EDS mapping images of $MoS_2/SnO_2$ QDs@CC.

**Figure S6.** CV plots of $MoS_2/SnO_2$ QDs@CC (a) and CC (b) collected at different scanning rates in 1 M $Na_2SO_4$; Relations of the current density and the scanning rate (c).

**Figure S7.** Contact angles of water on CC and $MoS_2/SnO_2$ QDs@CC.

**Figure S8.** Comparison of transmission (a) and absorption (b) for the CC and $MoS_2/SnO_2$ QDs @CC.

**Figure S9.** Digital images of $MoS_2/SnO_2$ QDs@CC (left) and CC (right) under dark (a) and illuminated states for 0.5 h (b), 1 h (c), 1.5 h (d) and 2 h (e).

**Figure S10**. Cycling performance of the battery at 500 mA $g^{-1}$ without optical window under alternative dark and illuminated states (a) and the corresponding GDC profiles (b).

**Figure S11.** CV curves of $MoS_2/SnO_2$ QDs@CC PRZIBs recorded at different rates of 0.2-1.0 mV $s^{-1}$ under dark (a) and illuminated (b) conditions; Linear relations of anodic peak currents ($i_p$) versus the square roots of scanning rate (c).

**Figure S12.** Determination of *b* values for cathodic and anodic peaks in dark (a) and illuminated (b) conditions; Comparison of capacitance capacity and diffusion-limited capacity contributions to anode current peak in photocathodes (c); CV profiles at 1.0 mV $s^{-1}$ showing the capacitive contribution (shaded area) to the total current under illuminated (d, top) and dark (d, bottom).

**Figure S13.** GCD profiles of $MoS_2/SnO_2$ QDs@CC PRZIBs obtained at current densities of 200 mA $g^{-1}$ (a), 500 mA $g^{-1}$ (b) and 1000 mA $g^{-1}$ (c) under dark and illuminated conditions.

**Figure S14.** Electrochemical impedance spectra of $MoS_2/SnO_2$ QDs@CC PRZIB under dark and illuminated conditions.

**Figure S15.** The DFT-optimized structures of the isolated $MoS_2$ bilayer and the isolated $SnO_2$ surface (a); $Zn^{2+}$ ion migration on the $MoS_2/SnO_2$ and the isolated $MoS_2$ bilayer (b).

**Figure S16.** Atomic ratios of Mo, S, Zn elements for $MoS_2/SnO_2$ QDs@CC at selected charge/discharge states.

**Figure S17.** Image showing the open-circuit voltage of a quasi-solid-state PRZIB assembled with $MoS_2/SnO_2$ QDs@CC (left); The GCD curves of a flexible quasi-solid-state Zn ion battery at 0.5 mA $cm^{-2}$ under dark and illuminated conditions (right).

**Figure S18.** Images showing the smart watch powered by a wearable wristband consisting of four series-connected QSSPZs in different bending radius of (a) 4, (b) 5, (c) 6 cm.

**Table S1.** Comparison of current variations of photocathode in different device configurations.

**Table S2.** Comparison of electrochemical performance of different photo-rechargeable battery systems under dark and illuminated conditions.

**Table S3.** Comparison of photoconversion efficiency of different photocathodes in

different photo-rechargeable batteries system.

## 3. Reference

# 1.Experimental Section

### *1.1 Material preparation*

**Preparation of $SnO_2$ QDs@CC substrates:** 1.2 g of stannous chloride dihydrate ($SnCl_2 \cdot 2H_2O$, Sigma-Aldrich) and 0.4 g of thiourea ($CH_4N_2S$, Sigma-Aldrich) were dissolved in 40 mL deionized water and stirred vigorously for 48 hours at room temperature, and then a clear and light-yellow solution containing $SnO_2$ QDs was obtained. After that, a piece of acid-treated CC ($4 \times 4 \times 0.036$ $cm^3$) was totally immersed into the $SnO_2$ QDs solution and finally annealed at 200 °C for 4 h under a vacuum condition.

**Preparation of $MoS_2$/$SnO_2$ QDs@CC photocathodes:** The as-obtained $SnO_2$ QDs@CC substrates were transferred to 30 mL aqueous $MoS_2$ precursor solution consisting of ammonium molybdate tetrahydrate (0.076 g, Sigma-Aldrich) and thiourea (1 g, Sigma-Aldrich). Subsequently, the precursor solution with substrates was transferred to an autoclave reactor and then kept at 180 °C for 15 h. Finally, the substrates were cleaned with deionized water and afterward dried at 80 °C.

### *1.2 Material characterization*

Morphologies and crystal structures of photocathodes were characterized by SEM (ZEISS Ultra 55), energy dispersive X-ray spectroscopy (EDS) and TEM (FEI Talos F200X). The composition and crystal structure of photocathode were determined by X-ray powder diffraction XRD (Rigaku, Cu Kα) with diffraction angles (2θ) ranging from 5 to 80°. The XPS experiments were performed by the surface analysis system (AXIS SUPRA) with Al Kα radiation to determine the elemental valence and bonding of the materials. The Raman spectra were recorded by using Alpha 300R Raman system with an excitation laser wavelength of 488 nm. The optical absorbance of the

photocathodes was tested by UV-vis (UV-759). The water contact angles were tested by Optical contact angle measuring instrument (JC2000D3P). The level of heat absorption of the material in illumination is evaluated by an infrared thermal imager (FOTRIC 226s). Absorption and transmittance of visible light from the material is measured using an Ocean Optics (USB4000).

### *1.3 Manufacturing and electrochemical testing of photodetectors*

The electrical photo-responses of $MoS_2/SnO_2$ QDs@CC were studied by planar metal-semiconductor-metal (MSM) and stacked PDs, respectively. Herein, the slurry was composed of 5 mg active materials ($MoS_2$ powder dissolved in NMP solvent). Similarly, the $SnO_2$ QDs solution and $MoS_2$ slurry were sequentially spin-coated onto fluorine-doped tin oxide (FTO) glass substrates, then waited for drying and vapor-coated with silver metal on the top contacts to obtain stacked PDs. The current-time response was measured with and without bias voltage under dark and illuminated conditions, respectively. The I-t tests were performed with a Keithley 2440 test system for photoelectric response from -1 V to +1 V in both dark and illuminated conditions.

### *1.4 Assembly of PRZIBs*

First, the commercial positive caps of coin cell (CR2032) were processed by cutting a 10 mm hole and then sealing the glass window with EVO-STIK epoxy resin, so that the PRZIBs could get illumination for operation. Thereafter, Zn foil anode, photocathode and glass microfiber separator (Whatman GF/B) were assembled in CR2032-type PRZIBs with 3 M Zn $(CF_3SO_3)_2$ aqueous electrolyte.

### *1.5 Assembly of flexible pouch cells*

The poly (vinyl alcohol) (PVA) polymer film was prepared by completely dissolving 3.5 g of PVA (Mw: ~89000-98000) in 50 mL DI water at 80 °C and naturally drying for 1 day. Subsequently, a saturated PVA/ Zn $(CF_3SO_3)_2$ gel electrolyte was obtained

via immersing the as-prepared PVA film in 3 M Zn $(CF_3SO_3)_2$electrolyte for 30 s. The QSSPZs was assembled by photocathode (2×2.5 $cm^2$), saturated PVA/ Zn $(CF_3SO_3)_2$ gel electrolyte and Zn foil anode (2×2.5 $cm^2$), which was encapsulated in aluminum plastic film (3×3.5 $cm^2$).

### *1.6 Electrochemical test of PRZIBs*

All PRZIB cyclic voltammetry (CV) cycles, voltage-time (I-t) tests, and AC impedance (EIS) measurements are measured using the CHIE660 electrochemical station. The impedance tests were performed over a frequency range of 100 kHz to 0.01 Hz with a voltage amplitude of 0.01 mV. The galvanostatic charge/discharge (GDC) profiles were performed on the Neware battery testing system. For the illuminated condition, Xenon lamp light source with wavelengths of 400−1100 nm, and intensity of 1 sun (100 mW $cm^{-2}$) was used.

### *1.7 Wearable demonstration*

The commercial smart-watch with Android system was used to construct the integrated self-powered smart watch devices. Before the operations, the battery in the smart watch has been removed. The four QSSPZs (size 1.0 × 4.0 cm ×0.2 cm) are charged to 1.3 V and connected in series. Finally, the QSSPZs are attached to drive the smart watch. All the heart rate information was sent to the mobile phone and cloud storage through Bluetooth in real time. The experimenter is informed and agrees to the public release of these experimental data.

### *1.8 DFT calculation*

Theoretical calculations were performed by Quantum ESPRESSO package[1]. We employed the ultrasoft pseudopotentials[2] with the Perdew−Burke−Ernzerhof (PBE) exchange−correlation functional[3] to optimize all the structures. The plane-wave kinetic energy cutoffs for the wavefunctions and the augmented charge density were

set to 25 and 250 Ry, respectively. To reduce computational cost, the $MoS_2/SnO_2$ heterojunction is created by placing a ($2 \times 5$) five atomic layers stoichiometric $SnO_2$ (001) surface onto a ($3\sqrt{2} \times 4\sqrt{2}$) $MoS_2$ bilayer (lattice mismatch: ~ 4%). There are 294 atoms in the heterojunction. A vacuum layer of ~15 Å was applied to eliminate the spurious interaction between the periodic images. The Grimme's DFT-D2 method[4] was applied to consider the weak van der Waals interactions between $MoS_2$ bilayer and $SnO_2$. Due to the large size of the $MoS_2/SnO_2$ heterojunction, only the Gamma point was sampled in the Brillouin zone in the calculations. The activation energy of ion diffusion was computed by the climbing image nudged elastic band (CI–NEB) method[5]. We calculate the binding energy of the $MoS_2/SnO_2$ by the following expressions: $E_b = E(MoS_2/SnO_2) + E(SnO_2) - E(MoS_2)$, where $E(MoS_2/SnO_2)$, $E(SnO_2)$ and $E(MoS_2)$ represents the total energy of the $MoS_2/SnO_2$ heterojunction, the isolated $MoS_2$ bilayer, the isolated $SnO_2$ surface, respectively. The $Zn^{2+}$ adsorption energy is calculated by the by the following expressions: $\Delta E = E_{(Zn/slab)} + E_{(Zn)} - E_{(slab)}$, where $E_{(Zn/slab)}$, $E_{(Zn)}$ and $E_{(slab)}$ represents the total energy of the $Zn^{2+}$ on the heterojunction (or $MoS_2$), $Zn^{2+}$, the heterojunction (or $MoS_2$), respectively.

## 2. Supplementary Figures and Tables

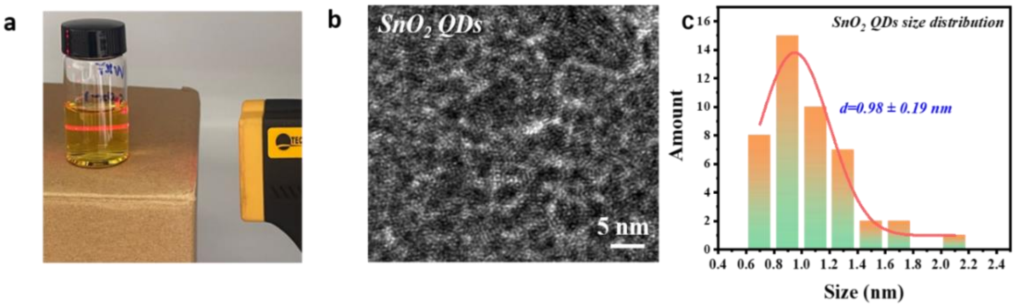


**Figure S1.** (a) Digital graph of a laser beam irradiated $SnO_2$ QDs solution; (b) HRTEM image and (c) particle size distribution of $SnO_2$ QDs.

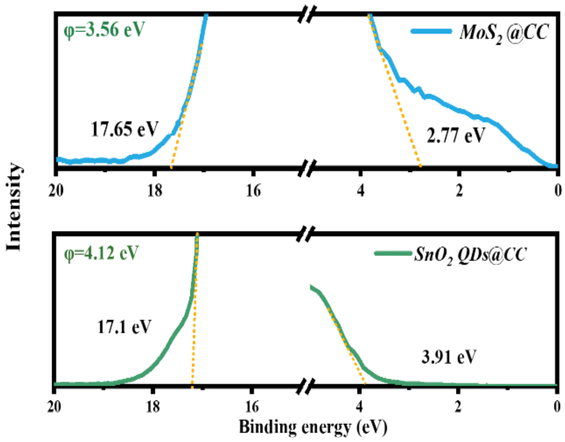


**Figure S2.** UPS curves of $MoS_2$@CC and $SnO_2$ QDs@CC.

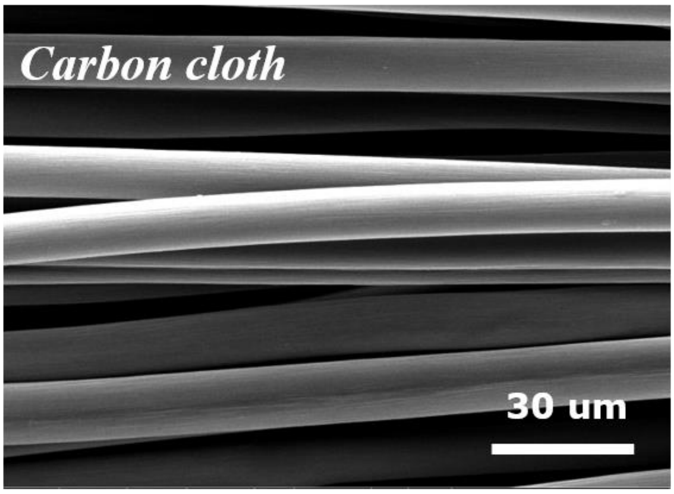


**Figure S3.** SEM image of carbon cloth.

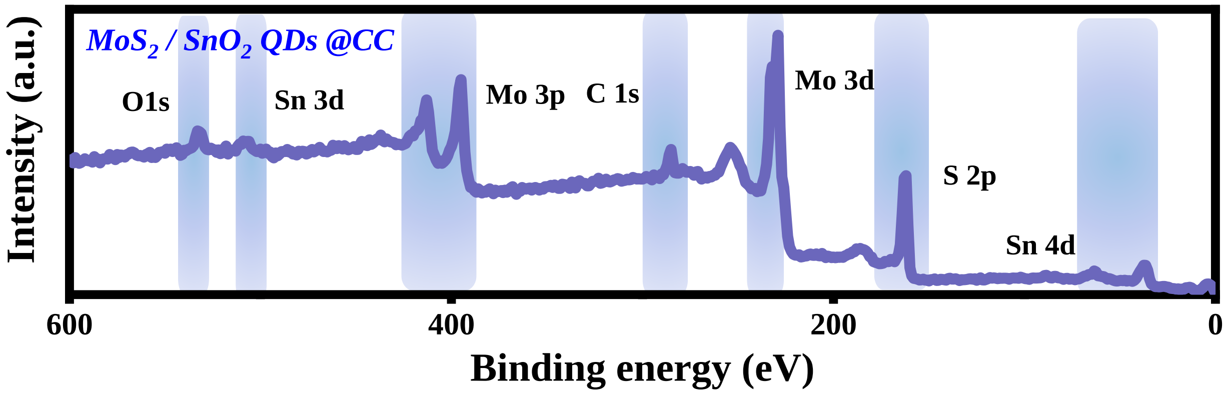


**Figure S4.** Full XPS spectra of $MoS_2/SnO_2$ QDs@CC.

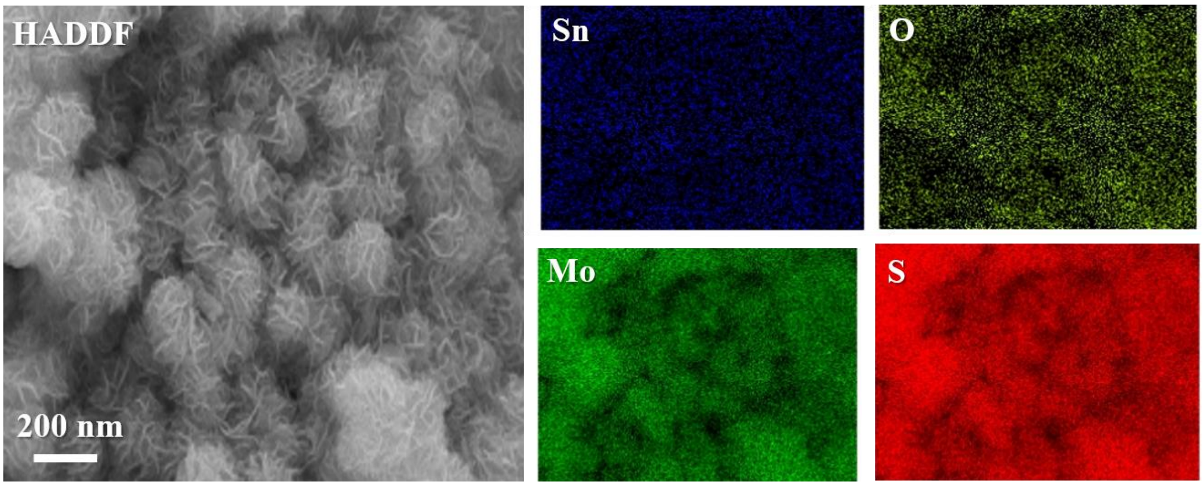


**Figure S5.** EDS mapping images of $MoS_2/SnO_2$ QDs@CC.

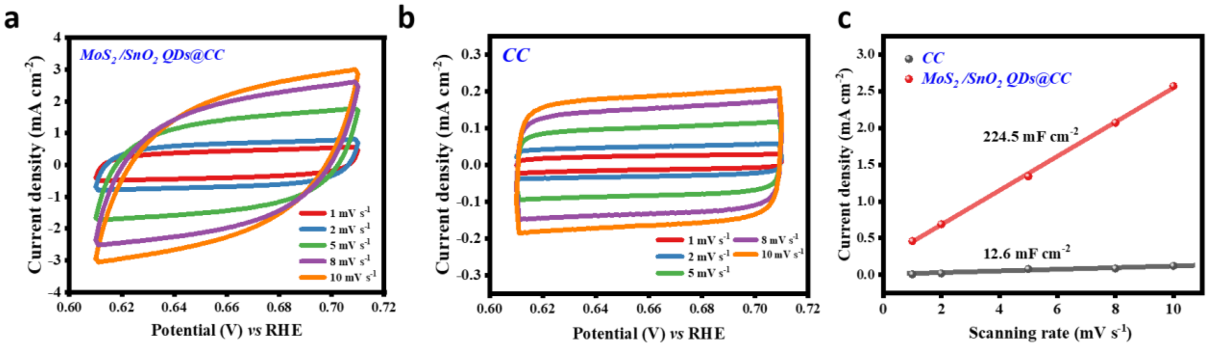


**Figure S6.** CV plots of (a) $MoS_2/SnO_2$ QDs@CC and (b) CC collected at different scanning rates in 1 M $Na_2SO_4$; (c) Relations of the current density and the scanning rate.

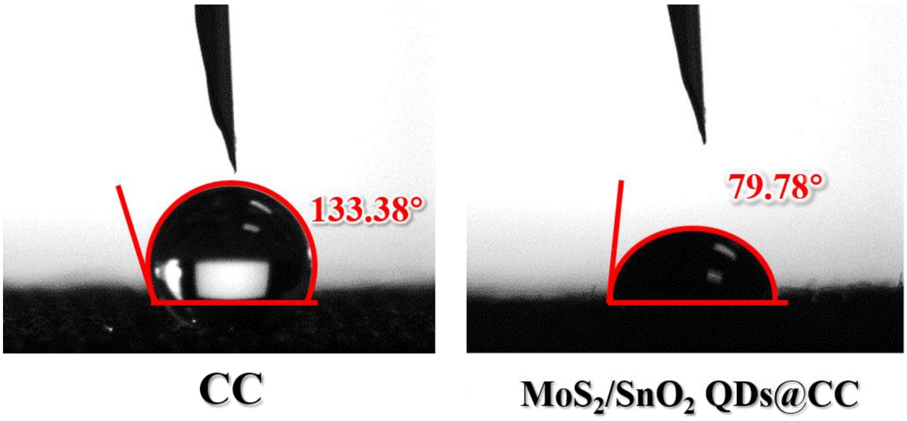


**Figure S7.** Contact angles of water on CC (left) and $MoS_2/SnO_2$ QDs@CC (right).

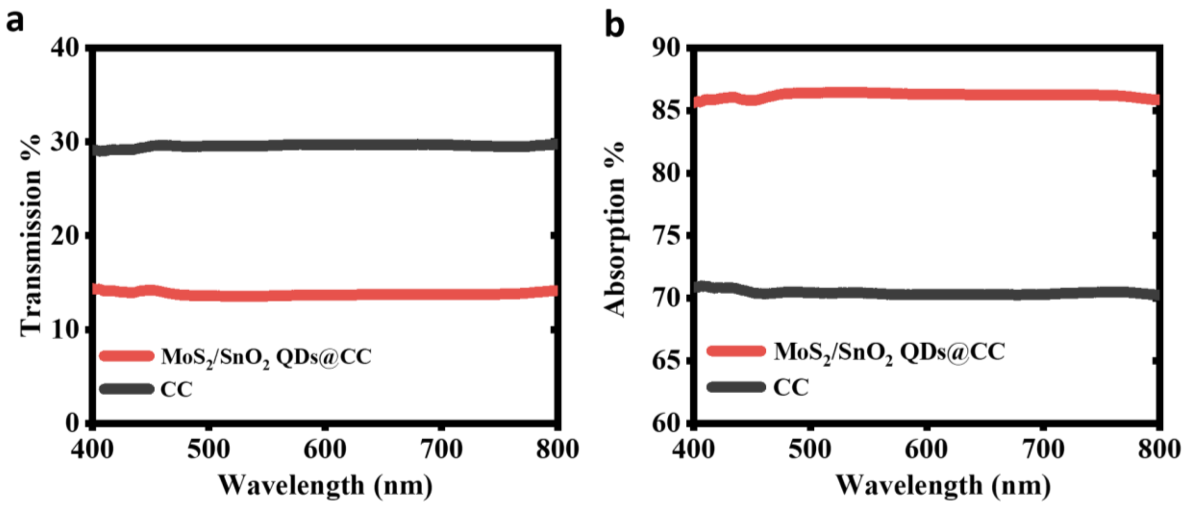


**Figure S8.** Comparison of (a) transmission and (b) absorption for CC and $MoS_2/SnO_2$ QDs @CC.

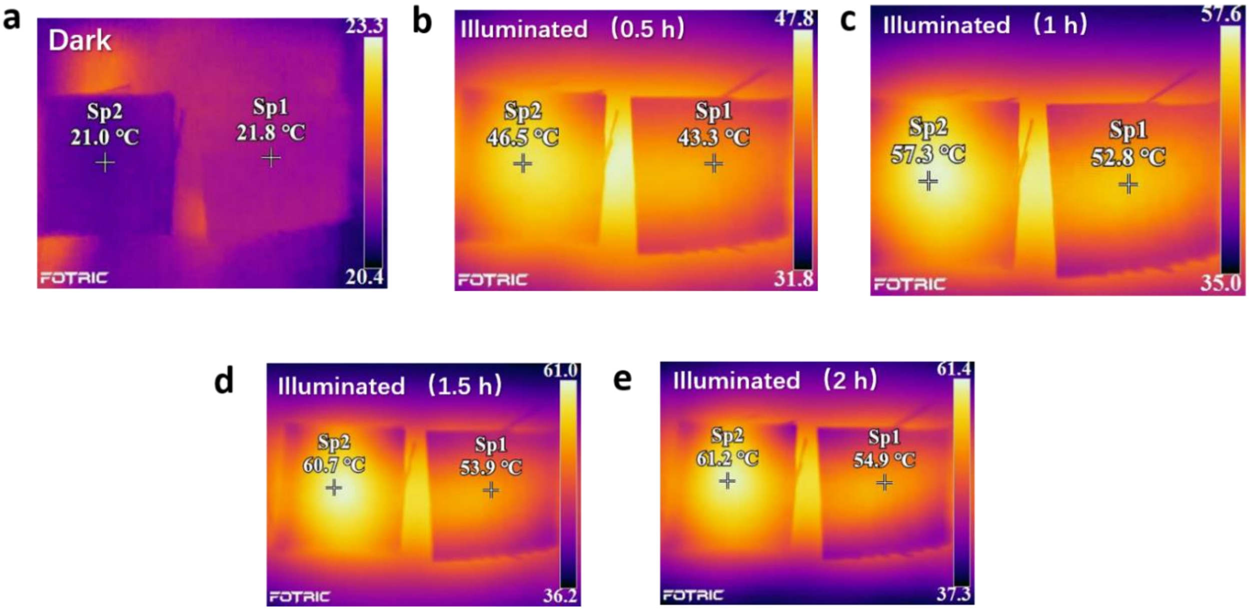


**Figure S9.** Digital images of $MoS_2/SnO_2$ QDs@CC (left) and CC (right) under (a) dark and illuminated states for (b) 0.5 h, (c) 1 h, (d) 1.5 h and (e) 2 h.

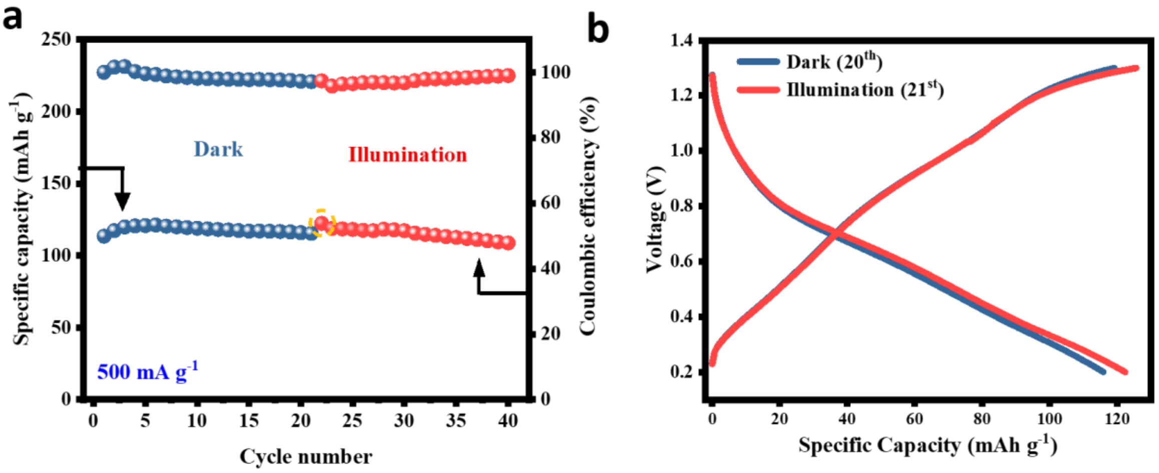


**Figure S10**. Cycling performance of the battery at 500 mA $g^{-1}$ without optical window under alternative dark and illuminated states (a) and the corresponding GDC profiles (b).

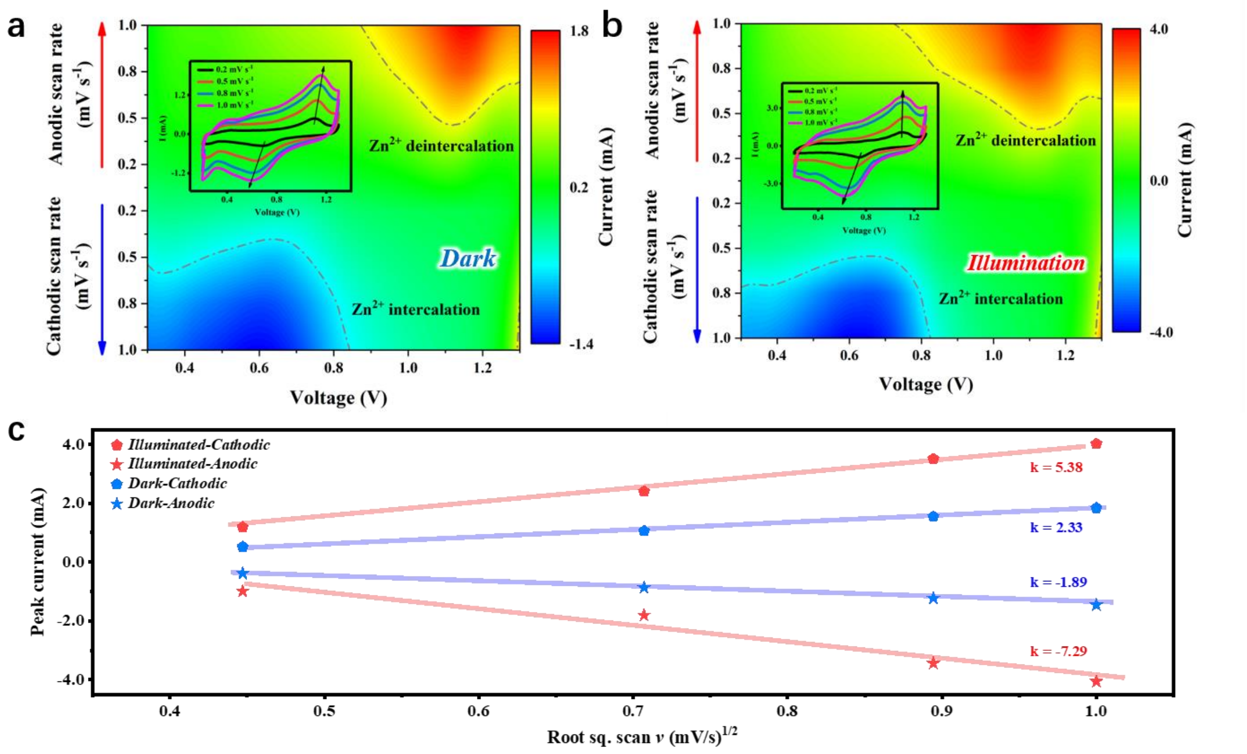


**Figure S11.** CV curves of $MoS_2/SnO_2$ QDs@CC PRZIBs recorded at different rates of 0.2-1.0 mV $s^{-1}$ under (a) dark and (b) illuminated conditions; Linear relations of anodic peak currents ($i_p$) versus the square roots of scanning rate (c).

**Note**: The $Zn^{2+}$ diffusion coefficient ($D$) is extracted by the Randles–Sevcik equation[6]

$$\boldsymbol{i_p = 2.69 \times 10^5 n^{3/2} AD^{1/2} Cv^{1/2}} \qquad \textbf{(eq.S1)}$$

where $i_p$ refers to the peak current, $n$ is the number of electrons in the reaction, $A$ is the electrode area, $C$ is the concentration of $Zn^{2+}$ in the electrolyte, and $v$ is the scanning rate.

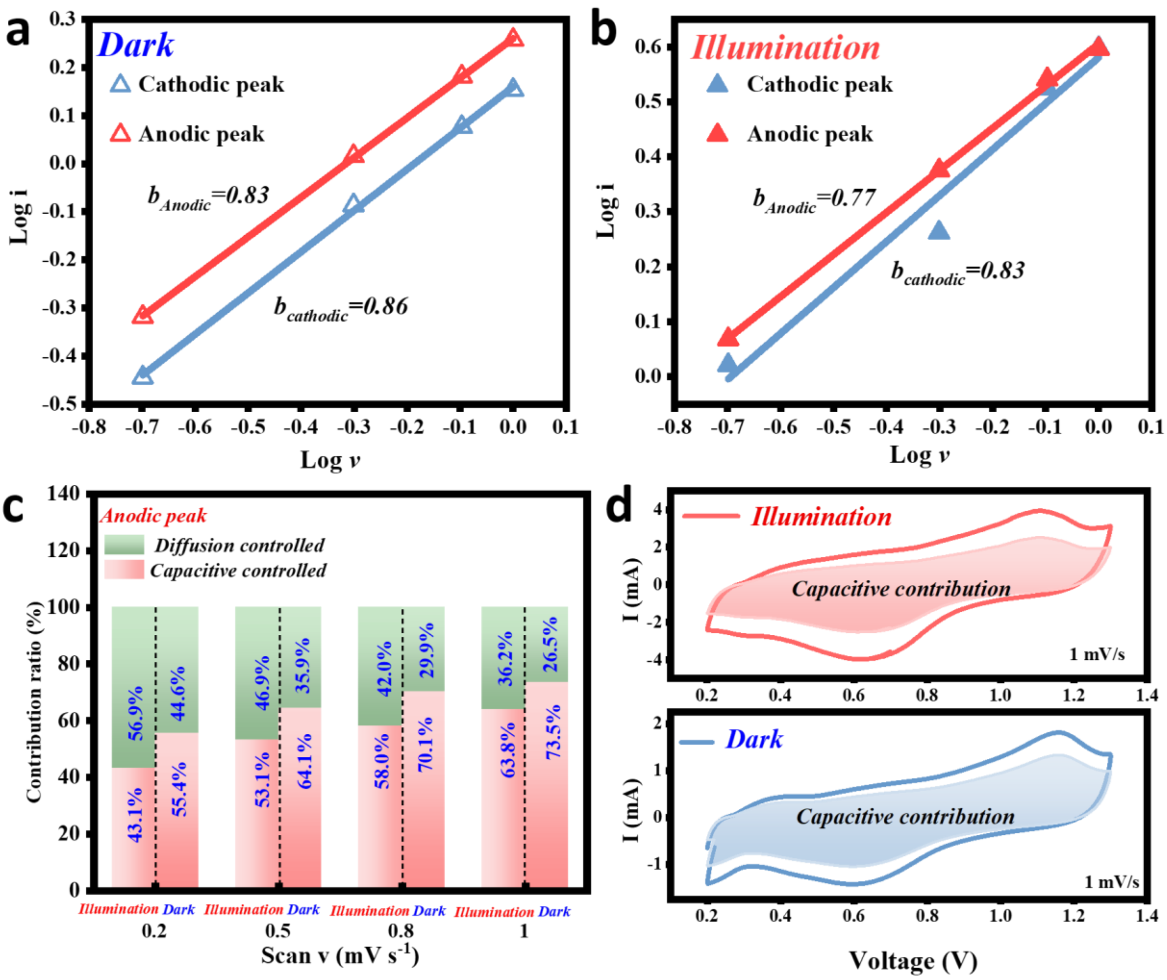


**Figure S12.** Determination of *b* values for cathodic and anodic peaks in dark (a) and illuminated (b) conditions; Comparison of capacitance capacity and diffusion-limited capacity contributions to anode current peak in photocathodes (c); CV profiles at 1.0 mV s$^{-1}$ showing the capacitive contribution (shaded area) to the total current under illuminated (d, top) and dark (d, bottom).

**Note**:

$$i = i_{diff} + i_{cap} = av^{b} \quad \text{or} \quad \log(i)=\log(a)+b\times\log(v) \qquad \text{(eq.S2)}$$

where, $i_{diff}$ represents diffusion-limited current, $i_{cap}$ is capacitive-limited current, and ***a*** and ***b*** are adjustable parameters. The charge storage mechanism is closely related to ***b*** value. If ***b*** is ~0.5, the charge storage is dominated by diffusion processes, while if ***b*** is between 0.5~1, the process is controlled by pseudo-capacitance behavior.[7]

The charge storage contributions can be split into capacitive-controlled (**$k_1\nu$**) and diffusion-controlled (**$k_2\nu^{1/2}$**) parts as a function of the voltage and can be expressed by **eq. 3**.[8]

$$i(\mathrm{V}) = k_1\nu + k_2\nu^{1/2} \text{ or } i(\mathrm{V})\nu^{-1/2} = k_1\nu^{1/2} + k_2 \qquad \text{(eq.S3)}$$

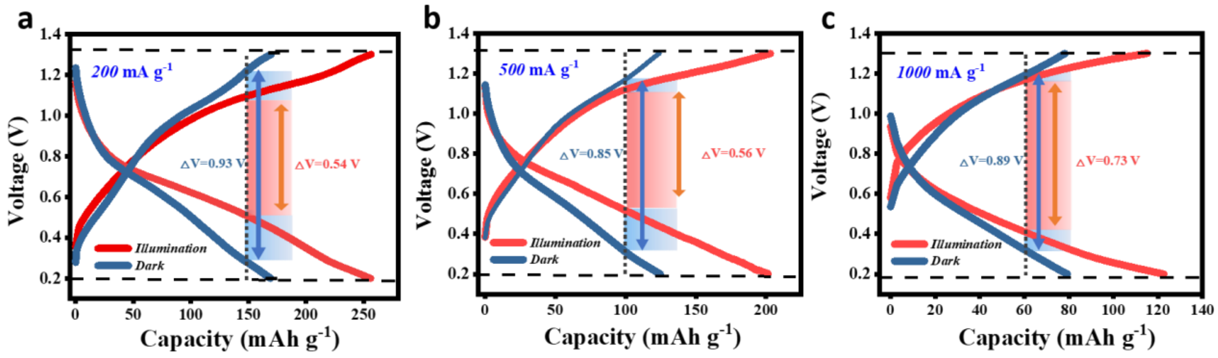


**Figure S13**. GCD profiles of $MoS_2/SnO_2$ QDs@CC PRZIBs obtained at current densities of (a) 200 mA $g^{-1}$, (b) 500 mA $g^{-1}$ and (c) 1000 mA $g^{-1}$ under dark and illuminated conditions.

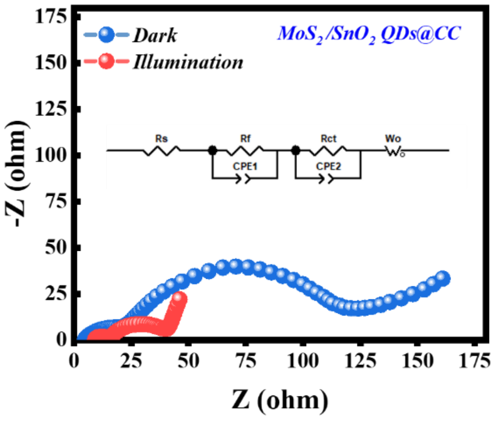


**Figure S14.** Electrochemical impedance spectra of $MoS_2/SnO_2$ QDs@CC PRZIB under dark and illuminated conditions.

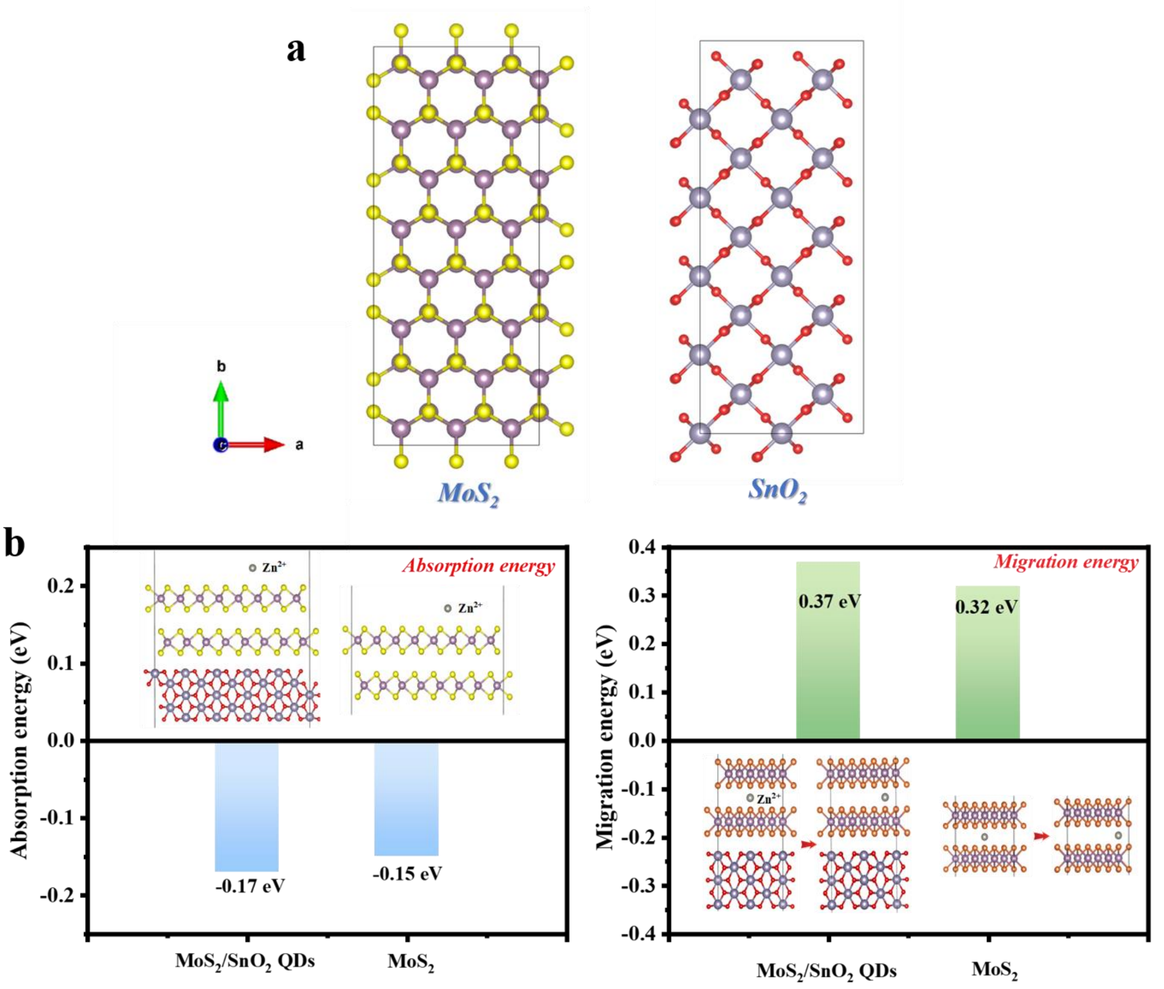


**Figure S15.** (a) DFT-optimized structures of the isolated $MoS_2$ bilayer and the isolated $SnO_2$ surface; (b) Absorption and migration energy with $Zn^{2+}$ on the $MoS_2/SnO_2$ and the isolated $MoS_2$ bilayer.

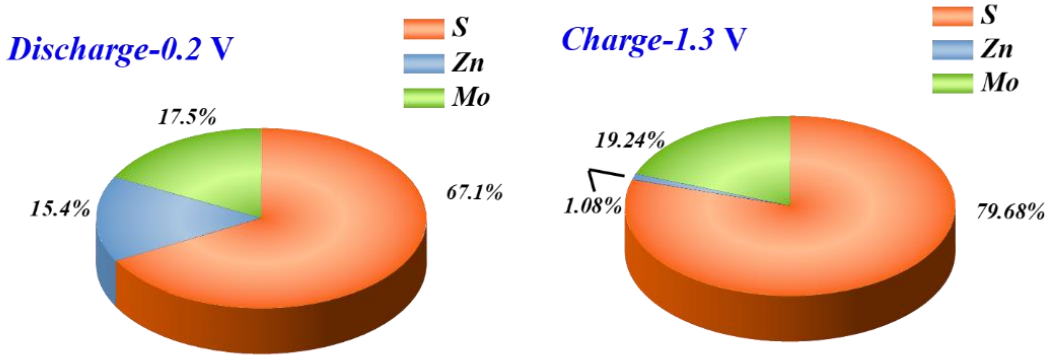


**Figure S16.** Atomic ratios of Mo, S, Zn elements for $MoS_2/SnO_2$ QDs@CC at selected charge/discharge states.

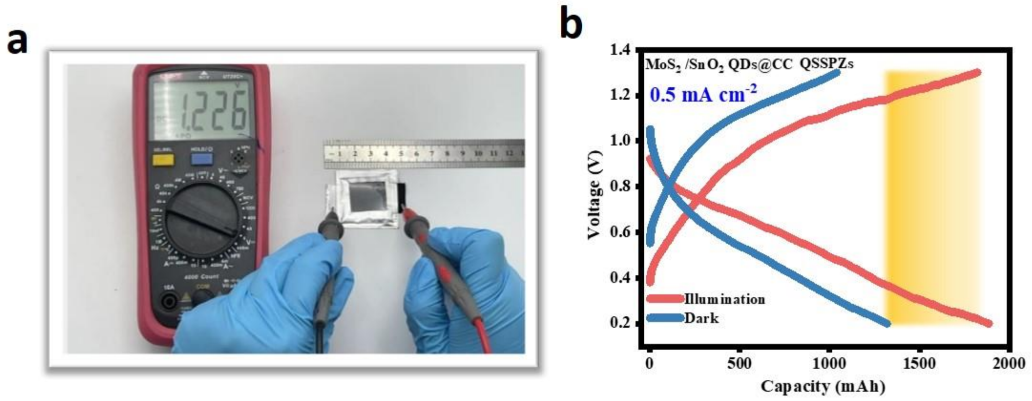


**Figure S17.** Image showing open-circuit voltage of a QSSPZ assembled with $MoS_2/SnO_2$ QDs@CC (a), and GCD curves of the QSSPZ at a current density of 0.5 mA cm$^{-2}$ under dark and illuminated conditions (b).

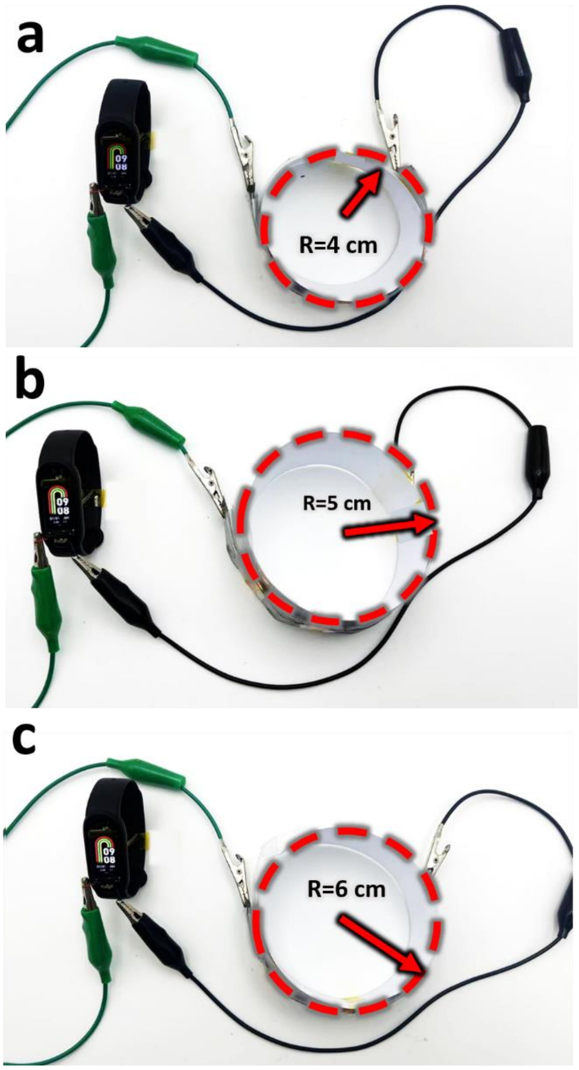


**Figure S18.** Images showing the smart watch powered by a wearable wristband consisting of four series-connected QSSPZs in different bending radius of (a) 4, (b) 5, (c) 6 cm.

**Table S1.** Comparison of current variations of photocathode in different device configurations.

| Photocathode | Configuration | Current variation ($\|\triangle I\|=I_{illu}-I_{dark}$) | Ref. |
|---|---|---|---|
| $VO_2$ | Au-$VO_2$-Au | 1.4 uA($V_{bias}$=1 V) | [9] |
| $VO_2$/rGO | FTO/rGO/$VO_2$/Ag | 4.4 uA | [10] |
| | Photoelectrode//Zn | 47 uA | |
| $V_2O_5$ | Au-$V_2O_5$-Au | 11 uA ($V_{bias}$=1 V) | [11] |
| rGO/P3HT/$V_2O_5$ | FTO/rGO/P3HT/$V_2O_5$/Ag | 65 nA ) | [12] |
| | FTO/ P3HT/ $V_2O_5$/ rGO/Ag | 0.68 uA | |
| | Photoelectrode//Zn | 9.5 uA | |
| | Photoelectrode//Li | 0.67 uA | |
| $MoS_2$ | Au-$MoS_2$-Au | 1.4 uA($V_{bias}$=0.1 V) | [13] |
| $MoS_2$/ZnO | FTO/ZnO/$MoS_2$/Ag | 1.75 uA | |
| $TiO_2$@$MoS_2$ HSs@NC | Au-$TiO_2$@$MoS_2$ HSs@NC-Au | 3.25 mA ($V_{bias}$=1V) | [14] |
| | FTO/$TiO_2$@$MoS_2$ HSs@NC/Ag | 1 uA | |
| $MoS_2$ NR | $MoS_2$ NR/AgCl/Ag/C three electrode | 1.8 uA $cm^{-2}$ | [15] |
| $TiS_2$–$TiO_2$ NSs | Photoelectrode//Li | 0.1 mA $cm^{-2}$ | [16] |
| $MoS_2$ | Au-$MoS_2$-Au | 3 uA ($V_{bias}$=1 V) | this work |
| $MoS_2$/$SnO_2$ QDs | FTO/$SnO_2$ QDs/$MoS_2$/Ag | 2.6 uA | this work |
| | Photoelectrode//Zn | 190 uA | this work |

**Table S2.** Comparison of electrochemical performance of different photo-

rechargeable battery systems under dark and illuminated conditions.

| Photocathode | Type | Current density (mA $g^{-1}$) | Capacity in dark (mAh $g^{-1}$) | Capacity in illumination (mAh $g^{-1}$) | Increased capacity | Ref. |
|---|---|---|---|---|---|---|
| $VO_2$/rGO | ZIBs | 200 | 282 | 315 | 11.7% | [9] |
| | | 20000 | 71 | 134 | 88.7% | |
| $V_2O_5$ | ZIBs | 50 | 190 | 370 | 94.7% | [12] |
| | | 1000 | 103 | 137 | 33% | |
| | LIBs | 200 | 118 | 161 | 36.4% | [11] |
| | | 500 | 81 | 127 | 56.8% | |
| $MoS_2$/ZnO | ZIBs | 100 | 245 | 340 | 38.8% | [13] |
| $TiO_2$@$MoS_2$ HSs@NC | SIBs | 200 | 525.5 | 650.4 | 23.8% | [14] |
| $MoS_2$/$SnO_2$ QDs | ZIBs | 100 | 190 | 366 | 92.6% | this work |

**Table S3.** Comparison of photoconversion efficiency of different photocathodes in

different photo-rechargeable batteries systems.

| Photocathode | Type | Photoconversion efficiency | Current density | Illuminated intensity ($mW\ cm^{-2}$) | Ref. |
|---|---|---|---|---|---|
| $VO_2$/rGO | ZIBs | 0.18% | 0.05 mA $cm^{-2}$ | 12* | [10] |
| rGO/P3HT/$V_2O_5$ | ZIBs | 1.2% | 100 mA $m^{-2}$ | 12 | [12] |
| g-$C_3N_4$@rGO | ZICs | 0.01% | - | 50* | [17] |
| Ag@$V_2O_5$ | ZICs | 0.05% | - | 50 | [18] |
| $MoS_2$/ZnO | ZIBs | 1.8%<br>0.2% | 0.025 mA $cm^{-2}$ | 12<br>100 | [13] |
| $VO_2$/ZnO | ZIBs | 0.51% | 0.02 mA $cm^{-2}$ | 12 | [9] |
| $TiO_2$@$MoS_2$ HSs@NC | SIBs | 0.71% | 2 mA $g^{-1}$ | 100 | [14] |
| $Cs_3Bi_2I_9$ | LIBs | 0.43% | 100 mA $g^{-1}$ | 100 | [19] |
| $MoS_2$/$MoO_x$ NR | LIBs | 0.05% | 0.2 mA $cm^{-2}$ | 100 | [15] |
| $TiS_2$/$TiO_2$ | LIBs | 0.23% | 0.12 mA $cm^{-2}$ | 70 | [16] |
| rGO/P3HT/$V_2O_5$ | LIBs | 2.6%<br>0.22% | 200 mA $m^{-2}$ | 12<br>100 | [12] |
| **$MoS_2$/$SnO_2$ QDs** | **ZIBs** | **2.7%**<br>**1.4%**<br>**0.79%** | **0.125 mA $cm^{-2}$**<br>**0.25 mA $cm^{-2}$**<br>**0.5 mA $cm^{-2}$** | **100** | **this work** |

* The light intensities of 12, 50, 70 and 100 mW $cm^{-2}$ correspond to light sources with wavelengths of 455 nm, 420 nm, white light, and 1 sun respectively.

## Reference

[1] P. Giannozzi, S. Baroni, N. Bonini, M. Calandra, R. Car, C. Cavazzoni, D. Ceresoli, G. L. Chiarotti, M. Cococcioni, I. Dabo, A. Dal Corso, S. de Gironcoli, S. Fabris, G. Fratesi, R. Gebauer, U. Gerstmann, C. Gougoussis, A. Kokalj, M. Lazzeri, L. Martin-Samos, N. Marzari, F. Mauri, R. Mazzarello, S. Paolini, A. Pasquarello, L. Paulatto, C. Sbraccia, S. Scandolo, G. Sclauzero, A. P. Seitsonen, A. Smogunov, P. Umari, R. M. Wentzcovitch, *J. Phys.: Condens. Matter* **2009**, 21, 395502.
[2] D. Vanderbilt, *Phys. Rev. B* **1990**, 41, 7892.
[3] J. P. Perdew, K. Burke, M. Ernzerhof, *Phys. Rev. Lett.* **1996**, 77, 3865.
[4] S. Grimme, *J. Comput. Chem.* **2006**, 27, 1787.
[5] G. Henkelman, H. J├│nsson, *J. Chem. Phys.* **2000**, 113, 9978.
[6] Denis Y. W. Yu, Christopher Fietzek, Wolfgang Weydanz, Kazunori Donoue, Takao Inoue, Hiroshi Kurokawa, Shin Fujitani, *J. Electrochem. Soc.* **2007**, 154, 4.
[7] V. Augustyn, J. Come, M. A. Lowe, J. W. Kim, P.-L. Taberna, S. H. Tolbert, H. D. Abruña, P. Simon, B. Dunn, *Nat. Mater.* **2013**, 12, 518.
[8] J. P. John Wang, James Lim, and Bruce Dunn*, *J. Phys. Chem. C* **2007**, 111, 6.
[9] B. Deka Boruah, M. De Volder, *J. Mater. Chem. A* **2021**, 9, 23199.
[10] B. Deka Boruah, A. Mathieson, S. K. Park, X. Zhang, B. Wen, L. Tan, A. Boies, M. De Volder, *Adv.Energy Mater.* **2021**, 11, 2100115.
[11] B. D. Boruah, B. Wen, M. De Volder, *Nano Lett.* **2021**, 21, 3527.
[12] B. D. Boruah, A. Mathieson, B. Wen, S. Feldmann, W. M. Dose, M. De Volder, *Energy Environ. Sci.* **2020**, 13, 2414.
[13] B. D. Boruah, B. Wen, M. De Volder, *ACS Nano* **2021**, 15, 16616.
[14] J. Li, Y. Zhang, Y. Mao, Y. Zhao, D. Kan, K. Zhu, S. Chou, X. Zhang, C. Zhu, J. Ren, Y. Chen, *Angew.* **2023**, 62.
[15] A. Kumar, P. Thakur, R. Sharma, A. B. Puthirath, P. M. Ajayan, T. N. Narayanan, *Small* **2021**, 17, 2105029.
[16] A. Kumar, R. Hammad, M. Pahuja, R. Arenal, K. Ghosh, S. Ghosh, T. N. Narayanan, *Small* **2023**, 19, 2303319.
[17] B. D. Boruah, A. Mathieson, B. Wen, C. Jo, F. Deschler, M. De Volder, *Nano Lett.* **2020**, 20, 5967.
[18] B. D. Boruah, B. Wen, S. Nagane, X. Zhang, S. D. Stranks, A. Boies, M. De Volder, *ACS Energy Lett.* **2020**, 5, 3132.
[19] N. Tewari, S. B. Shivarudraiah, J. E. Halpert, *Nano Lett.* **2021**, 21, 5578.
